# Effects of High-Intensity Interval Training and Moderate-Intensity Continuous Training on Cardiometabolic Risk Factors in Overweight and Obesity Children and Adolescents: A Meta-Analysis of Randomized Controlled Trials

**DOI:** 10.3390/ijerph182211905

**Published:** 2021-11-12

**Authors:** Meng Cao, Yucheng Tang, Shu Li, Yu Zou

**Affiliations:** 1 Department of Physical Education, Normal College, Shenzhen University, Shenzhen 518061, China; 2019128123@email.szu.edu.cn (Y.T.); 2020128225@email.szu.edu.cn (S.L.); 2 Department of Physical Education, Institute of KEEP Collaborative Innovation, Shenzhen University, Shenzhen 518061, China; 3 Department of Sport and Exercise Science, College of Education, Zhejiang University, Hangzhou 310058, China; zouyuzy@zju.edu.cn

**Keywords:** high-intensity interval training, cardiometabolic, obesity, children, adolescents

## Abstract

*Background*: The purpose of this review was to compare the effectiveness of high-intensity interval training (HIIT) and moderate-intensity continuous training (MICT) on cardiometabolic risk factors of obese children and adolescents. *Methods*: Relevant studies published in PubMed, MEDLINE and Web of Science databases were searched. Only randomized controlled trials (RCTs) that examined the effect of HIIT and MICT on children and adolescents with obesity were included. Meta-analyses were conducted to determine the effect of HIIT on cardiometabolic risk factors using STATA software and potential moderators were explored (i.e., study duration, training modalities, work/rest ratio and work duration time). *Results*: Twelve RCTs involving 325 participants were included in the meta-analysis. HIIT showed more positive effects on maximal oxygen uptake (VO_2max_; SMD = 0.87, 95% CI: 0.39 to 1.35, *p* = 0.000) and systolic blood pressure (SBP; SMD = −0.64, 95% CI: −1.05 to −0.22, *p* = 0.003) than MICT. However, when compared with MICT, HIIT caused no significant differences in body weight, body mass index, body fat percentage, diastolic blood pressure and glycolipid metabolism markers. Furthermore, subgroup analysis showed that the effects of HIIT on VO_2max_ and SBP were significantly different regarding protocol factors, such as modality, duration, training time, training settings, work/rest ratio and work duration. *Conclusions*: HIIT has a positive role in promoting cardiometabolic risk factors in obese children and adolescents. Moreover, when compared with MICT, HIIT had a more significant effect on improving cardiorespiratory fitness and systolic blood pressure. The factors of HIIT protocol had an important influence on the intervention effects of childhood obesity.

## 1. Introduction

Obesity is a chronic metabolic disease that is caused by the excessive accumulation of body fat, which causes physical and psychological harm [1]. In the past two decades, childhood obesity has reached epidemic proportions worldwide [2]. A recent epidemiological study showed that the number of obese children worldwide is about 107.7 million, and the rate of obesity in children is higher than that in adults [3]. Childhood obesity not only increases the risk of cardiovascular disease but also develops into diseases such as adult coronary heart disease, hypertension, metabolic syndrome and type II diabetes mellitus (T2DM) [4].

In 2020, the WHO’s (World health organization) latest guidelines on physical activity (PA) strongly recommended that children and adolescents aged 6–17 should do at least 60 min of moderate-to-vigorous physical activity (MVPA) every day, and resistance training at least three times per week to improve muscle strength [5]. In the 2012 Lancet physical activity report, nearly 80% of the 13–15-year-old adolescents failed to meet the current PA guidelines [6]. Accordingly, childhood and adolescence are critical periods for the development of physical fitness since physical inactivity is the main reason for the development of childhood obesity and related comorbidities [7]. Research has shown that a lack of time, insufficient motivation and poor adherence are common obstacles to physical inactivity [8]. High-intensity interval training (HIIT) may be a time-effective method for improving health indicators and is more suitable for children and adolescents in a natural state of high-intensity interval exercise patterns [9]. Furthermore, when compared with traditional endurance training or moderate-intensity continuous training (MICT), although HIIT has a lower training duration and volume, it can produce similar or even better impacts on health-related indicators [10].

Recent meta-analyses and systematic reviews of HIIT examined the effects of HIIT on body composition and cardiorespiratory fitness (CRF) in children and adolescents with obesity [10,11,12]. However, these systematic reviews and meta-analyses had the following limitations: (1) they only compared the effects between HIIT and MICT on body composition or CRF and did not include blood indicators, such as lipid profiles and glucose; (2) several recent and key studies were excluded. Moreover, the results of RCT studies on HIIT intervention in children and adolescents with obesity were inconsistent; HIIT is considered to improve cardiometabolic risk factors in obese children and adolescents [13,14,15], sometimes better than MICT on some indicators [16]. However, some studies did not observe an improvement due to HIIT [17] or showed that it had no better effects compared with MICT [18,19]. Other studies suggested that MICT has a greater effect on obese children and adolescents [20].

Therefore, the main objective of this meta-analysis was to conduct a comparative study on the effects of HIIT and MICT on cardiometabolic risk factors (such as body composition, CRF, blood pressure and glycolipid metabolism) in children and adolescents with obesity based on RCT studies. Furthermore, according to subgroup analysis, we aimed to examine whether the factors of HIIT protocols would affect the impact of interventions.

## 2. Methods

### 2.1. Search Strategy

This review’s registry is on PROSPERO (ID: CRD420183694). It references the PRISMA (Preferred Reporting Items for Systematic Reviews and Meta-Analysis) guidelines [21]. The literature of randomized controlled trials (RCTs) of HIIT intervention on cardiometabolic risk factors was searched, such as body mass (BM), body mass index (BMI), total body fat percentage (%BF), visceral adipose tissue (VAT), fat-free mass (FFM), maximal oxygen uptake (VO_2max_), systolic blood pressure (SBP), diastolic blood pressure (DBP), triglycerides (TG), total cholesterol (TC), high-density lipoprotein cholesterol (HDL), low-density lipoprotein cholesterol (LDL), blood glucose (BG), blood insulin (BI) and homeostasis model assessment (HOMA-IR). The following terms were used for literature retrieval in PubMed, MEDLINE and Web of Science until August 2021: (“high intensity interval” OR “high intensity intermittent” OR “aerobic interval training” OR “sprint interval” OR HIIT OR HIIE OR SIT) AND (obese OR obesity OR overweight) AND (child* OR adolescen* OR youth OR student* OR boy* OR girl* OR kid*). Additional relevant studies were searched according to the references that were included in the study.

### 2.2. Inclusion Criteria

Studies incorporating children and adolescents between 6 and 18 years were included. This included overweight/obese children and adolescents without disabilities. The inclusion criteria were as follows: (1) both HIIT and MICT training programs were included; (2) training intensity was defined as “all-out”, “maximal effort”, “≥90% VO_2max_” [22], “85~95% maximal heart rate” [23] or “≥100% maximal aerobic speed (MAS) [24]; (3) any of the outcomes included cardiometabolic risk factors (body composition, CRF, blood pressure and glycolipid metabolism indicators); (4) the study was available in English. Conference abstracts or unpublished articles were excluded.

### 2.3. Data Synthesis

One author (C.M.) conducted data extraction to extract the characteristics of the included studies, such as the year, author, country, number and characteristics of participants, gender, subject age range or mean, study duration, study settings, work/rest time ratio, training frequency, total HIIT time per session and per week and mean and standard deviation before and after training.

### 2.4. Risk of Bias

The risk of bias for studies that met the inclusion criteria was assessed by two independent reviewers (C.M. and T.Y.C). Discrepancies in the scores were resolved through consultation or via a third reviewer (L.S.). We assessed the risk of bias for the 12 studies according to the eight-item checklist of the modified PRISMA statement [12].

The risk of bias was assessed in each study based on an eight-item marked as ”clearly reported” (●) or ”not or unclearly reported” (○) for each of the following criteria: (1) qualification criteria were specified, (2) participants were randomly assigned, (3) there was no significant difference of the baseline values between groups of the main outcome(s), (4) blinding was used by assessors who measured the main outcome(s), (5) used ”intention to treat“ to analyze the primary outcome(s) data, (6) reported the dropout of main outcome(s) and the dropout of participants was <20%, (7) the sample size and the study had enough power to detect changes in the main outcome(s) and (8) reported the summary results of each group and estimated the effect size (difference between groups) and its precision (e.g., 95% confidence interval). The criteria were added to create an overall risk of bias score: high (0–3), moderate (4–6) and low (7–8).

### 2.5. Publication Bias

We used Egger’s and Begg’s statistic test to assess the publication bias, where *p* ≤ 0.05 is considered an existence bias [25]. The funnel plot was interpreted according to visual judgment, and the statistical bias of Egger’s test was used to confirm or refute publication bias. If there was a significant publication bias, the stability of the results was evaluated using a trim-and-fill method [26].

### 2.6. Statistical Analysis

Meta-analyses were conducted to determine the effect of HIIT on cardiometabolic risk factors in comparison to the moderate-intensity continuous training group (MICT). We used STATA software 14.0 for Windows (STATA 14.0, Stata Corp., College Station, TX, USA) to examine the mean values or change score and its standard deviations in the meta-analysis. The results of the meta-analysis with random effects are represented in the figures (the mixed effects are reported in the text). Heterogeneity was quantified using Cochrane’s Q test and Higgins I^2^, where <25, 50 and 75 represent low, moderate and high heterogeneities, respectively. Separate meta-analyses were carried out for: (1) cardiorespiratory fitness (estimated or actual VO_2max_), (2) body composition (body mass—BM, body mass index—BMI and body fat percentage and fat-free-mass—FFM), (3) blood pressure (SBP and DBP) and (4) glycolipid indicators (blood glucose—BG, blood insulin—BI, HOMA-IR, TG, TC, HDL-C and LDL-C). The standardized mean difference (SMD) and the 95% confidence intervals were reported. The significance level was set at *p* < 0.05. The subgroup moderator analyses were conducted when the HIIT effects differed according to the duration of training (i.e., <12 weeks vs. ≥12 weeks), training modality (i.e., running or cycling), work/rest time ratio (<1, =1 or > 1) and work duration time (<1 min, 1–4 min or 4 min). Moderator effects were considered statistically significant at *p* < 0.01.

## 3. Results

### 3.1. Included Studies

**Figure 1:**
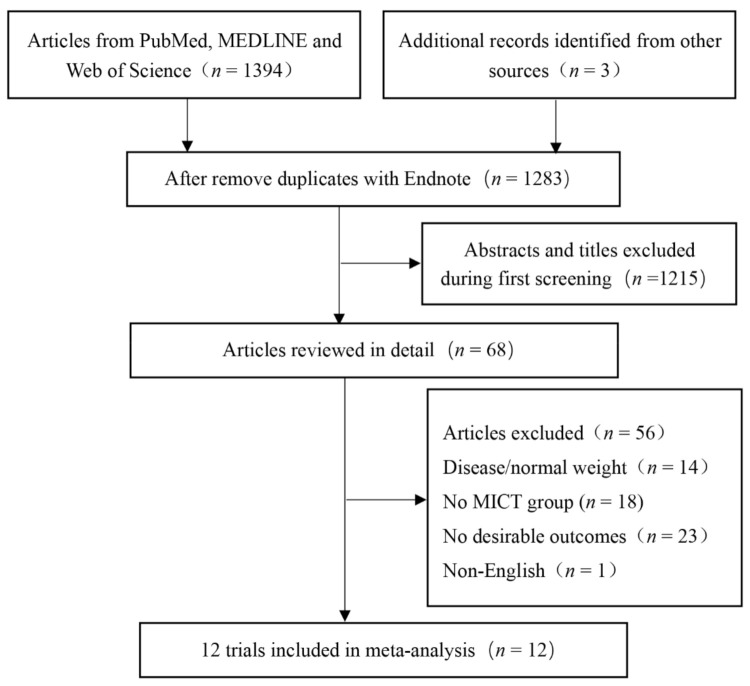
Flow diagram of the study selection. MICT: moderate-intensity continuous training.

Through electronic data retrieval, 1397 articles were found and 114 duplicate articles were excluded. Subsequently, 1215 studies inconsistent with the topics were excluded. In total, the full text of 68 studies were evaluated, and 56 studies were excluded due to the following reasons: disease/normal weight (*n* = 14), no MICT group (*n* = 18), no desirable outcomes (*n* = 23), and non-English (*n* = 1). After evaluation, we conducted a final meta-analysis of 12 RCTs that met the inclusion and exclusion criteria (Figure 1). The program characteristics for HIIT and MICT interventions are summarized in Table 1.

### 3.2. Results

In total, 325 overweight/obese children and adolescents were included in this study. The duration of training ranged from 3 to 24 weeks, and 13–60 children and adolescents in each study. Seven RCTs were from Europe, one from Asia, and the remaining four were from America. Five RCTs were from Europe, two each from North and South America, and one each from Asia, Africa, and Oceania. The average age of the children and adolescents that were included in this study ranged from 10.4 to 16.8 years old, and four trials only included males; two did not report the gender. We used the revised design of the PRISMA statement to evaluate the quality of the study [12] in which one trial had a low risk (score of 7–8), nine trials had a moderate risk (score of 4–6) and two trials had a high risk (score of 0–3) of bias (Table 2).

### 3.3. Body Composition

#### 3.3.1. Body Mass (BM)

According to the data of 11 trials that examined the effect of HIIT vs. MICT on BM, when compared with MICT, there were no significant differences due to HIIT on BM (SMD: −0.10; 95% CI: −0.61 to 0.41; *p* = 0.705; Figure 2). In addition, there was a high heterogeneity in the weight (I^2^ = 78.4%; *p* = 0.000). The sensitivity analysis was conducted by excluding each test in sequence, and the results were robust, indicating that there was no significant difference. No significant publication bias for weight was detected (*p*-value for Egger: 0.965; *p*-value for Begg: 0.644) (Supplementary Figures S1–S26).

#### 3.3.2. Body Mass Index (BMI)

According to the data of 12 trials that examined the effect of HIIT vs. MICT on BMI, no significant difference between HIIT and MICT for BMI was observed (SMD: 0.03; 95% CI: −0.75 to 0.80; *p* = 0.949; Figure 3). Moreover, high heterogeneity was detected for weight (I^2^ = 89.8%; *p* = 0.000). The sensitivity analysis was conducted by excluding each test in sequence, and the results were robust, indicating that there was no significant difference. No significant publication bias for weight was detected (*p*-value for Egger: 0.611; *p*-value for Begg: 0.537) (Supplementary Figures S1–S26).

#### 3.3.3. Body fat percentage (BF%)

According to the data of 10 trials that examined the effect of HIIT vs. MICT on BF%, no significant difference between HIIT and MICT for BF% was observed (SMD: −0.23; 95% CI: −0.73 to 0.28; *p* = 0.380; Figure 4). Moreover, moderate heterogeneity was detected for weight (I^2^ = 74.5%; *p* = 0.000). The sensitivity analysis was conducted by excluding each test in sequence, and the results were robust, indicating that there was no significant difference. No significant publication bias for weight was detected (*p*-value for Egger: 0.437; *p*-value for Begg: 0.283) (Supplementary Figures S1–S26).

#### 3.3.4. Fat-Free Mass (FFM)

The data of six trials were examined regarding the effect of HIIT and MICT on FFM; no significant difference between MICT and HIIT on FFM was found (SMD: 0.15; 95% CI: −0.16 to 0.45; *p* = 0.338; Figure 5). Moreover, low heterogeneity was detected for weight (I^2^ = 3.6%; *p* = 0.394). The sensitivity analysis was conducted by excluding each test in sequence, and the results were robust, indicating that there was no significant difference. No significant publication bias for weight was detected (*p*-value for Egger: 0.735; *p*-value for Begg: 0.707) (Supplementary Figures S1–S26).

#### 3.3.5. Abdominal Fat (AF)

The data of six trials were examined regarding the effect of HIIT vs. MICT on AF; no significant difference between HIIT and MICT for AF was observed (SMD: −0.21; 95% CI: −1.19 to 0.76; *p* = 0.670; Figure 6). Moreover, high heterogeneity was detected for weight (I^2^ = 88.3%; *p* = 0.000). The sensitivity analysis was conducted by excluding each test in sequence, and the results were robust, indicating that there was no significant difference. No significant publication bias for weight was found (*p*-value for Egger: 0.124; *p*-value for Begg: 1.000) (Supplementary Figures S1–S26).

### 3.4. Cardiorespiratory Fitness (CRF)

Eight trials were examined regarding the effect of HIIT vs. MICT on CRF (VO_2max_); HIIT’s significantly greater effects on VO_2max_ compared with MICT were observed (SMD: 0.87; 95% CI: 0.39 to 1.35; *p* = 0.000; Figure 7). Moreover, moderate heterogeneity was detected for weight (I^2^ = 56.8%; *p* = 0.023). The sensitivity analysis was conducted by excluding each test in sequence, and the results were robust, indicating that there was no significant difference. No significant publication bias for weight was detected (*p*-value for Egger: 0.377; *p*-value for Begg: 0.711) (Supplementary Figures S1–S26).

### 3.5. Blood Pressure

#### 3.5.1. Systolic Blood Pressure (SBP)

The data of seven trials regarding the effect of HIIT vs. MICT on SBP showed that, when compared with MICT, HIIT significantly decreased SBP (SMD: −0.64; 95% CI: −1.05 to −0.22; *p* = 0.003; Figure 8). Moreover, low heterogeneity was detected for weight (I^2^ = 44.9%; *p* = 0.092). A sensitivity analysis revealed a robust conclusion and showed a non-significant difference by sequentially excluding each trial (Supplementary Figures S1–S26). No significant publication bias for weight was detected (*p*-value for Egger: 0.192; *p*-value for Begg: 0.133; Supplementary Figures S16–S26). When the potential publication bias was adjusted by using the trim-and-fill method, the conclusion did not change (SMD: −0.40, 95% CI: −0.68 to −0.13, *p* = 0.004), and the funnel plot after shearing and supplementation showed no obvious asymmetry, suggesting no publication bias (Supplementary Figures S16–S26).

#### 3.5.2. Diastolic Blood Pressure (DBP)

The data of seven trials were examined regarding the effect of HIIT vs. MICT on DBP; no significant difference between HIIT and MICT was observed for DBP (SMD: −0.13; 95% CI: −0.88 to 0.61; *p* = 0.728; Figure 9). Moreover, low heterogeneity was detected for weight (I^2^ = 82.1%; *p* = 0.000). The sensitivity analysis was conducted by excluding each test in sequence, and the results were robust, indicating that there was no significant difference. No significant publication bias for weight was detected (*p*-value for Egger: 0.113; *p*-value for Begg: 0.072; Supplementary Figures S16–S26). When the potential publication bias was adjusted by using the trim-and-fill method, after filling two studies, the conclusion did not change (SMD: 0.32, 95% CI: −0.48 to 1.12, *p* = 0.429), and the funnel plot after shearing and supplementation showed no obvious asymmetry, suggesting no publication bias (Supplementary Figures S1–S26).

### 3.6. Lipid Metabolism Markers

#### 3.6.1. Triglycerides (TG)

The data of five trials were examined regarding the effect of HIIT vs. MICT on TG; no significant difference between HIIT and MICT was observed for TG (SMD: −0.20; 95% CI: −0.59 to 0.19; *p* = 0.321; Figure 10). Moreover, low heterogeneity was detected for weight (I^2^ = 33.2%; *p* = 0.200). A sensitivity analysis revealed a robust conclusion and showed a non-significant difference by sequentially excluding each trial (Supplementary Figures S1–S26). No significant publication bias for weight was detected (*p*-value for Egger: 0.198; *p*-value for Begg: 0.221; Supplementary Figures S16–S26). The funnel plot after shearing and supplementation showed no obvious asymmetry, suggesting no publication bias (Supplementary Figures S16–S26).

#### 3.6.2. Total Cholesterol (TC)

The data of five trials were examined regarding the effect of HIIT vs. MICT on TC; no significant difference between HIIT and MICT was observed for TC (SMD: −0.11; 95% CI: −0.94 to 0.72; *p* = 0.798; Figure 11). Moreover, low heterogeneity was detected for weight (I^2^ = 24.8%; *p* = 0.000). The sensitivity analysis was conducted by excluding each test in sequence, and the results were robust, indicating that there was no significant difference. No significant publication bias for weight was detected (*p*-value for Egger: 0.049; *p*-value for Begg: 0.462; Supplementary Figures S16–S26). The funnel plot after shearing and supplementation showed no obvious asymmetry, suggesting no publication bias (Supplementary Figures S1–S26).

#### 3.6.3. High-Density Lipoprotein-Cholesterol (HDL-C)

The data of four trials were examined regarding the effect of HIIT vs. MICT on HDL-C. When compared with MICT, HIIT demonstrated no significant differences for HDL-C (SMD: −0.18; 95% CI: −1.34 to 0.99; *p* = 0.764; Figure 12). Moreover, high heterogeneity was detected for weight (I^2^ = 89.5%; *p* = 0.000). The sensitivity analysis was conducted by excluding each test in sequence, and the results were robust, indicating that there was no significant difference. No significant publication bias for weight was detected (*p*-value for Egger: 0.060; *p*-value for Begg: 0.734; Supplementary Figures S16–S26). The funnel plot after shearing and supplementation showed no obvious asymmetry, suggesting no publication bias (Supplementary Figures S1–S26).

#### 3.6.4. Low-Density Lipoprotein Cholesterol (LDL-C)

The data of three trials were examined regarding the effect of HIIT vs. MICT on LDL-C; no significant difference between HIIT and MICT was observed for LDL-C (SMD: −0.01 95% CI: −1.19 to 1.18; *p* = 0.995; Figure 13). Moreover, high heterogeneity was detected for weight (I^2^ = 87.0%; *p* = 0.000). The sensitivity analysis was conducted by excluding each test in sequence, and the results were robust, indicating that there was no significant difference. No significant publication bias for weight was detected (*p*-value for Egger: 0.084; *p*-value for Begg: 0.296; Supplementary Figures S16–S26). The funnel plot after shearing and supplementation showed no obvious asymmetry, suggesting no publication bias (Supplementary Figures S1–S26).

### 3.7. Glucose Metabolism Markers

#### 3.7.1. Blood Glucose (BG)

The data of four trials were examined regarding the effect of HIIT versus MICT on BG. The analyzed results suggested that HIIT demonstrated no significant effect on BG when compared with MICT (SMD: −0.02; 95% CI: −0.37 to 0.34; *p* = 0.935; Figure 14), and low heterogeneity was detected (I^2^ = 0.0%; *p* = 0.406). A sensitivity analysis was conducted by excluding each test in sequence, and the results were robust, indicating that there was no significant difference. No significant publication bias was observed for BMI (*p*-value for Egger: 0.555; *p*-value for Begg: 0.734) (Supplementary Figures S1–S26).

#### 3.7.2. Blood Insulin (BI)

The data of four trials compared the effects of HIIT versus MICT on BI. The analyzed results showed that when compared with MICT, HIIT had no significant effects on BI (SMD: −0.21; 95% CI: −0.99 to 0.57; *p* = 0.596; Figure 15), and moderate heterogeneity was detected (I^2^ = 74.5%; *p* = 0.008). A sensitivity analysis was conducted by excluding each test in sequence, and the results were robust, indicating that there was no significant difference. No significant publication bias was observed for BMI (*p*-value for Egger: 0.250; *p*-value for Begg: 0.734) (Supplementary Figures S1–S26).

#### 3.7.3. HOMA-IR

The data of four trials were examined regarding the effect of HIIT versus MICT on BI. The pooled results suggested that HIIT demonstrated no significant effect on BI when compared with MICT (SMD: 0.08; 95% CI: −0.54 to 0.70; *p* = 0.803; Figure 16), and moderate heterogeneity was detected (I^2^ = 70.1%; *p* = 0.008). A sensitivity analysis was conducted by excluding each test in sequence, and the results were robust, indicating that there was no significant difference. No significant publication bias was observed for BMI (*p*-value for Egger: 0.026; *p*-value for Begg: 0.086) (Supplementary Figures S1–S26).

### 3.8. Subgroup Analysis

#### 3.8.1. Subgroup Analysis of HIIT on Cardiorespiratory Fitness

The subgroup analysis was based on the training parameters of the HIIT program, such as the training modality, duration, time, settings, work/rest time ratio and work duration. The subgroup analysis results demonstrated that the modality, duration, training time, training settings, work/rest time ratio and work duration were key parameters that were associated with VO_2max_ improvement. HIIT protocol of > 8 weeks (SMD = 1.05, 95% CI: 0.34 to 1.76, *p* = 0.043), running (SMD = 1.05, 95% CI: 0.47 to 1.64, *p* = 0.024), work/rest ratio > 1 (SMD = 0.75, 95% CI: 0.36 to 1.14, *p* = 0.391), work duration <1 min/= 4 min (SMD = 1.38, 95%CI: 0.10 to 2.66, *p* = 0.009 or SMD = 0.62, 95%CI: 0.08 to 1.17, *p* = 0.446) and each training time <20 min (SMD = 1.64, 95% CI: 0.79 to 2.49, *p* = 0.218) had a better effect on improving the VO_2max_ of obese children and adolescents compared with MICT (Table 3).

#### 3.8.2. Subgroup Analysis of HIIT on Systolic Blood Pressure

Another subgroup analysis demonstrated that a HIIT protocol > 8 weeks (SMD = −0.63, 95% CI: −1.11 to −0.15, *p* = 0.056), running (SMD = −0.73, 95% CI: −1.16 to −0.31, *p* = 0.286), work/rest ratio < 1 (SMD = −0.93, 95% CI: −1.46 to −0.40, *p* = 0.843), work duration <1 min (SMD = −0.96, 95% CI: −1.65 to −0.26, *p* = 0.149) and each training time >30 min (SMD = −0.82, 95% CI: −1.40 to −0.24, *p* = 0.092) had a better effect on improving the SBP of obese boys younger than 13 years (SMD = −0.58, 95% CI: −0.98 to −0.18, *p* = 0.722) (Table 4).

## 4. Discussion

The main findings of the present study revealed that HIIT significantly improved VO_2max_ and SBP compared with MICT (Table 5). When compared with MICT, HIIT showed no significant difference regarding BM, BMI, AF, FFM, DBP, TG, TC, HDL, LDL, BG, BI and HOMA-IR. Furthermore, a subgroup analysis showed that VO_2max_ and SBP were significantly different in different subgroups, such as modality, duration, training time, training settings, work/rest ratio and work duration.

### 4.1. Body Composition

Body composition, especially fat content, is an important index that affects the health of the obese population. The main purpose of a weight control program is to reduce body fat and improve body composition. This study showed that although there is no significant difference between HIIT and MICT in improving body composition, both training protocols can effectively reduce body composition indexes, such as BMI and body fat percentage. Our results are consistent with a previous review by Batacan et al. [34], which synthesized 65 studies and showed that HIIT could significantly improve the waist circumference and body fat percentage of overweight or obese populations. Another systematic review from Wewege et al. also showed that HIIT and MICT had similar effects on improving body composition in overweight or obese adults [35]. In addition, although HIIT and MICT have no difference in improving body composition, the physiological mechanisms of HIIT and MICT improving body composition are different. Moderate-intensity exercise may involve an increase in the fat burning rate as a matrix, with a sustained high release of free fatty acids (FFAs) and subsequent oxidation of FFAs, whereas the potential mechanisms of HIIT in reducing fat include an increase in catecholamines after exercise, which improve fat oxidation and the decomposition of visceral adipose tissue, a decrease in appetite and an increase in excess post-exercise oxygen consumption after HIIT [36,37].

### 4.2. Cardiorespiratory Fitness

Cardiorespiratory fitness (CRF) is the ability of the body to absorb oxygen and transport it to skeletal muscle to provide energy for physical activities; it has been identified as a powerful predictor of cardiometabolic disease outcomes in children and adolescents [38]. VO_2max_ is the gold standard for evaluating CRF. This study demonstrated that both HIIT and MICT could effectively improve VO_2max_ in obese children and adolescents, and HIIT was better than MICT; this further expanded the previous findings [39]. The subgroup analysis of this study also showed that the positive effects of HIIT on VO_2max_ were different when changing the training modality, duration, time, settings, work/rest ratio and work duration. Studies showed that obese children and adolescents have significantly lower CRF than normal-weight peers, which increases the cardiovascular disease risk [40]. In addition, the level of CRF will also affect the mental health and well-being of children and adolescents (i.e., self-esteem and depression). Therefore, CRF is a variable that can strongly predict the health of children and adolescents. Increasing VO_2max_ through exercise is particularly important for them. The effect of HIIT on improving CRF is better than MICT, which may depend on the factors that affect oxygen delivery and extraction, including cardiac output (e.g., heart rate and stroke volume), peripheral perfusion and diffusion ability and skeletal muscle oxidation ability [41,42]. In addition, HIIT improves VO_2max_ better than MICT, where one of the mechanisms may be that HIIT increases the mitochondrial oxidation capacity. [43,44]. Importantly, although our results indicate that running may provide more health benefits than cycling; when obese children engage in HIIT, we should be concerned that the increase in joint torque and ground reaction forces may increase the risk of joint degeneration in obese children and adolescents.

### 4.3. Blood Pressure

Hypertension (HBP) is one of the main risk factors of cardiovascular disease that is induced by childhood obesity [45]. Some studies have shown that childhood HBP will develop into adulthood and positively correlates with an increased risk of organ injury, such as coronary artery calcification, heart ventricle hypertrophy and increased carotid intima-media thickness [46]. Obesity was shown to be the main cause of HBP in children and adolescents. Due to the rapidly increased prevalence of obese children and adolescents in China, the population attributable risk (PAR%) of HBP steadily increased from 6.3% in 1995 to 19.2% in 2014 [47,48]. Seven studies that were included in our review compared the effects of HIIT versus MICT on blood pressure; the results showed that HIIT significantly improved SBP in obese children and adolescents when compared with MICT, while showing no significant difference for DBP. This is inconsistent with the findings for adults; the results from Batacan [34] and Costa [49] showed that there was no significant difference between HIIT and MICT regarding improving SBP.

Studies showed that a reduction in SBP can reduce the risk of cardiovascular disease and mortality. An SBP decrease by 5 mmHg reduces stroke mortality by 14%, reduces coronary heart disease mortality by 9% and reduces all-cause mortality by 7% [50]. High-intensity increased blood flow velocity, elevated nitric oxide (NO) level in endothelial cells and increased nitric oxide is dependent on peripheral vascular compliance, which may be a potential mechanism for HIIT to reduce blood pressure [51,52]. The mechanisms by which exercise lowers blood pressure are complex and not fully understood. Studies have shown that MICT can also decrease DBP [53], which may be why there was no significant difference between HIIT and MICT in our study. Compared with MICT, HIIT can significantly decrease SBP and its physiological mechanisms include not only the relevant adaptation of NO but also promotes an increase in brachial artery diameter [54]. In addition, Cornelissen et al. observed that a decrease in SBP during the day was associated with a greater increase in VO_2max_ [53]. It was well established that HIIT has superior benefits on CRF when compared with MICT, which is consistent with the results of this study.

### 4.4. Glycolipid Metabolism

Strong evidence indicated that obesity is often accompanied by disorders of glycolipid metabolism, such as insulin resistance and dyslipidemia [55]. It is also an important inducement of cardiovascular disease (CVD) and T2DM [56]. Studies showed that disorders of glycolipid metabolism may originate from childhood; obesity can accelerate this situation [57]. Therefore, strategies to improve glycolipid metabolism in children and adolescents with obesity play an important role in disease prevention. This study showed that HIIT could improve the glycolipid metabolism markers of obese children and adolescents, but there was no difference that was attributable to MICT. A recent meta-analysis found that short-term HIIT (≤12 weeks) significantly decreased the fasting glucose of the overweight/obese population, but had no effects on the lipid profiles [34]. The mechanism of HIIT’s improvement of glucose metabolism remains to be explored. The increased translocation of GLUT-4 to the plasma membrane and the activation of AMP-activated kinase (AMPK) in skeletal muscle may be its potential mechanism. [58]. In addition, compared with moderate-intensity exercise, high-intensity exercise can recruit a larger proportion of muscle fibers, which may explain the improvement of glucose metabolism regulation after HIIT. The mechanisms of MICT improving glycolipid metabolism may be different. Presently, there are the following potential mechanisms: MICT increases skeletal muscle GLUT-4 expression and increases sarcolemma glucose transport, promoting the improvement of glucose metabolism. However, the association between increased glucose uptake and GLUT-4 translocation is still controversial [59]. MICT favors fatty acid oxidation, limits hepatic triglyceride accumulation, and impairs the detrimental actions of fatty acid derivatives in the insulin receptor signaling cascade; it may be another potential mechanism for MICT to improve glycolipid metabolism [60].

### 4.5. Strengths and Limitations

This study has the following strengths: (1) This meta-analysis compared comprehensive cardiometabolic outcomes between HIIT and MICT in children and adolescents with obesity. (2) The analysis of this study was based on the results of randomized controlled trials with high-quality evidence. In addition to strengths, this study also has the following limitations: (1) There were relatively few studies on some indicators; more relevant studies are needed to expand the results in the future. (2) This study only included published RCT studies, and publication bias will still affect the comprehensiveness of the data to a certain extent. Larger sample sizes and more diverse studies are needed to address these limitations.

## 5. Conclusions

In conclusion, our study demonstrated that HIIT had a positive role in promoting cardiometabolic risk factors in obese children and adolescents, and suggested that HIIT had better effects on cardiorespiratory fitness and systolic blood pressure in childhood obesity. In addition, the factors of an HIIT protocol, such as modality, training duration, time, and work/rest ratio, affected the training effects. Our results suggested that HIIT can be an effective alternative to MICT for maintaining cardiometabolic health in obese children and adolescents.

## Figures and Tables

**Figure 2:**
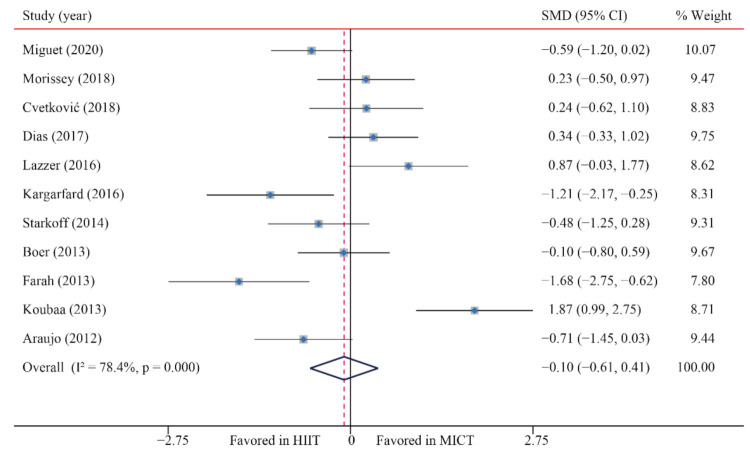
Effect of HIIT versus MICT on body mass. SMD: Standardized mean difference, CI: Confidence interval.

**Figure 3:**
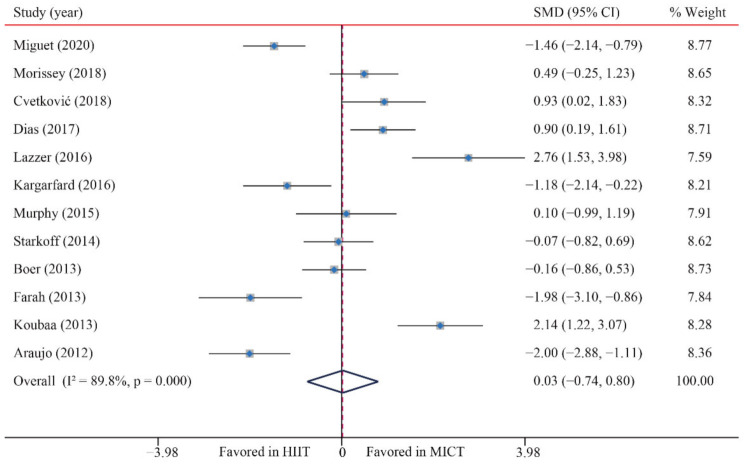
Effect of HIIT versus MICT on body mass index.

**Figure 4:**
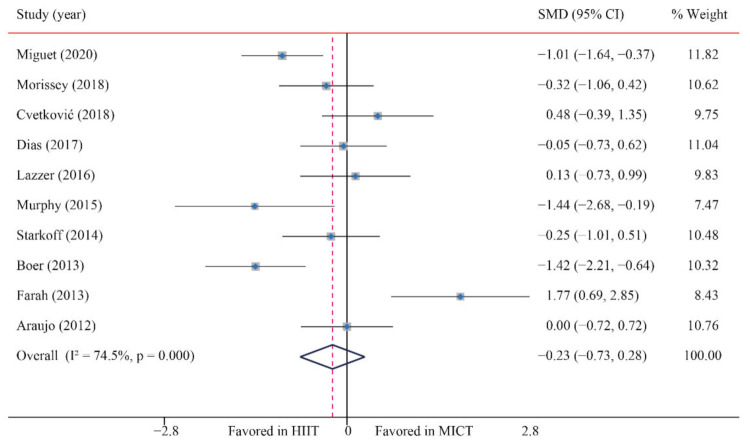
Effect of HIIT versus MICT on body fat percentage.

**Figure 5:**
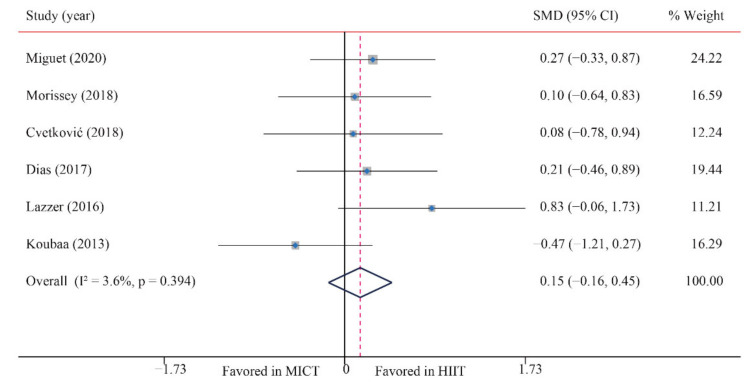
Effect of HIIT versus MICT on fat free mass.

**Figure 6:**
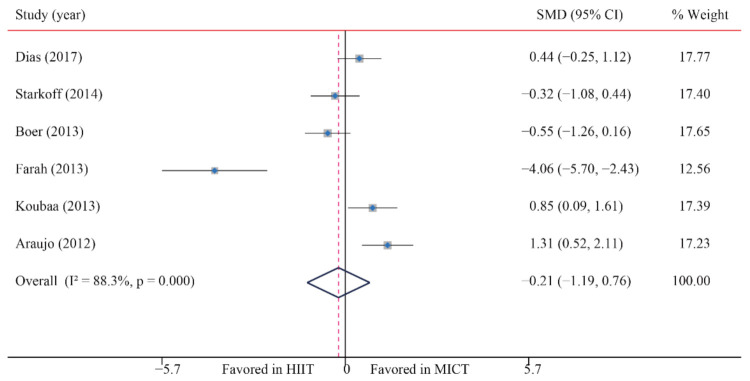
Effect of HIIT versus MICT on abdominal fat.

**Figure 7:**
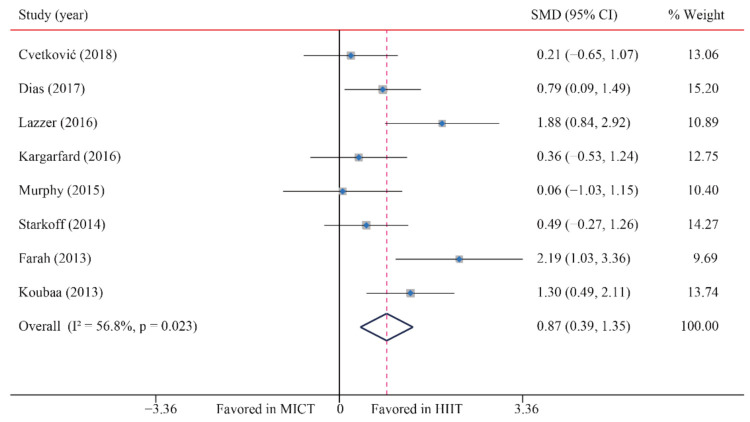
Effect of HIIT versus MICT on maximal oxygen uptake.

**Figure 8:**
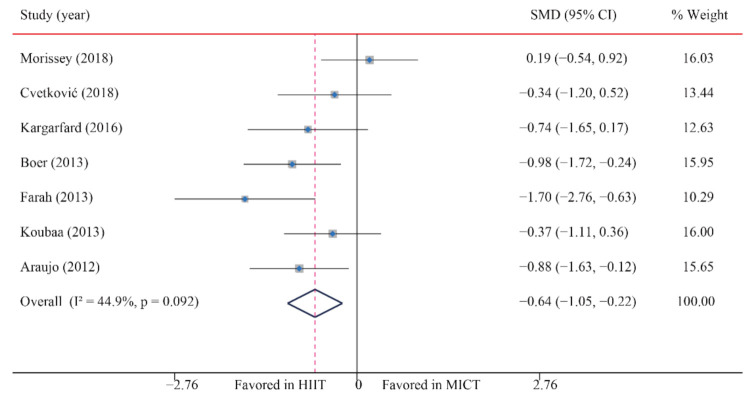
Effect of HIIT versus MICT on systolic blood pressure.

**Figure 9:**
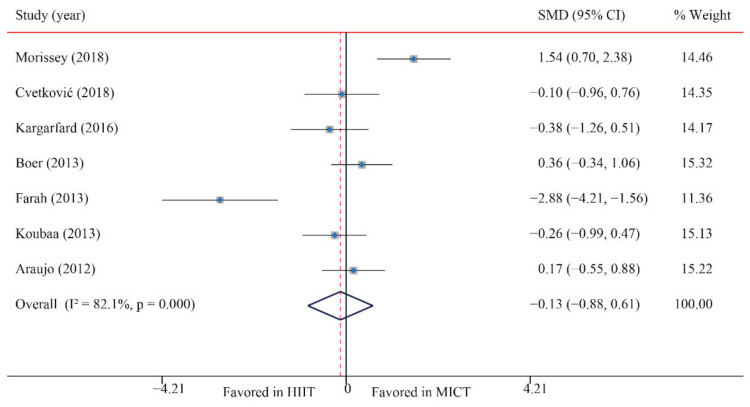
Effect of HIIT versus MICT on diastolic blood pressure.

**Figure 10:**
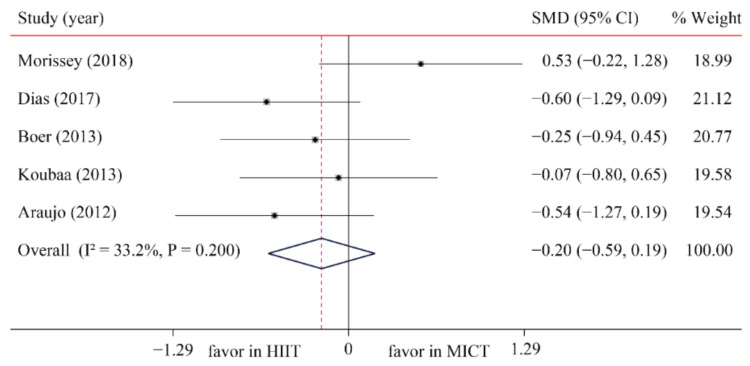
Effect of HIIT versus MICT on triglycerides.

**Figure 11:**
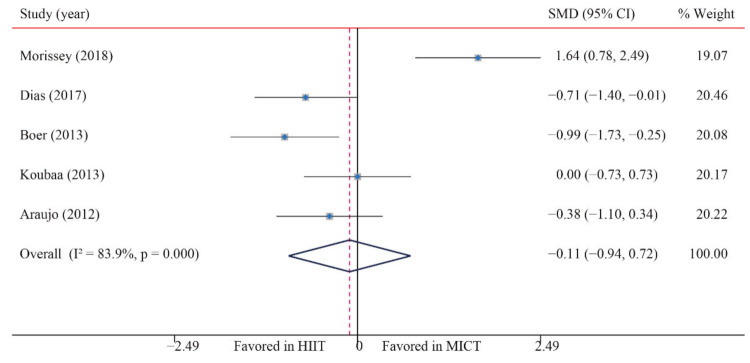
Effect of HIIT versus MICT on total cholesterol.

**Figure 12:**
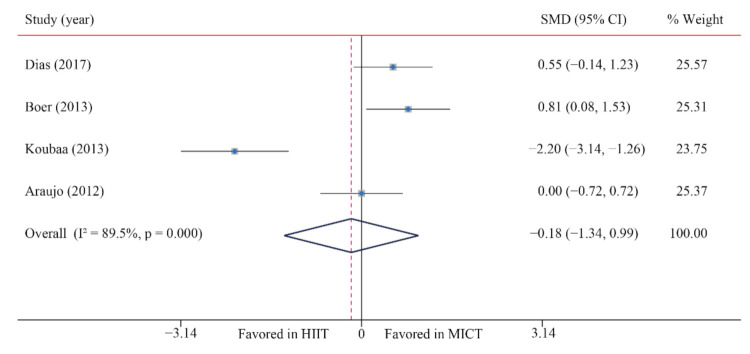
Effect of HIIT versus MICT on high-density lipoprotein cholesterol.

**Figure 13:**
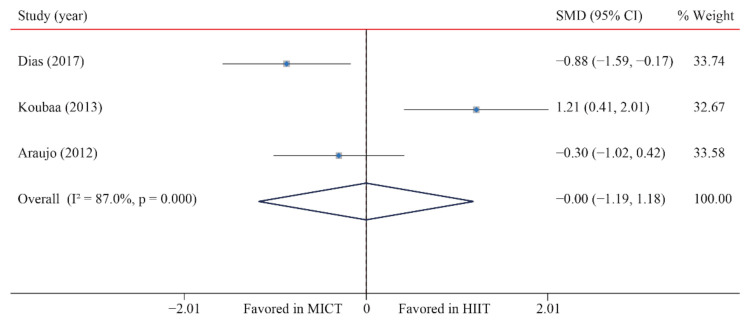
Effect of HIIT versus MICT on low-density lipoprotein cholesterol.

**Figure 14:**
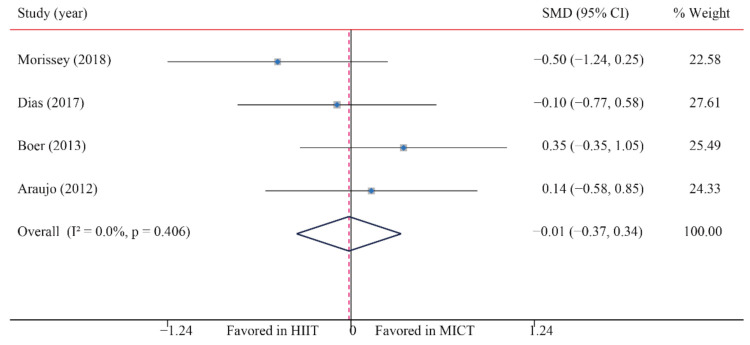
Effect of HIIT versus MICT on blood glucose.

**Figure 15:**
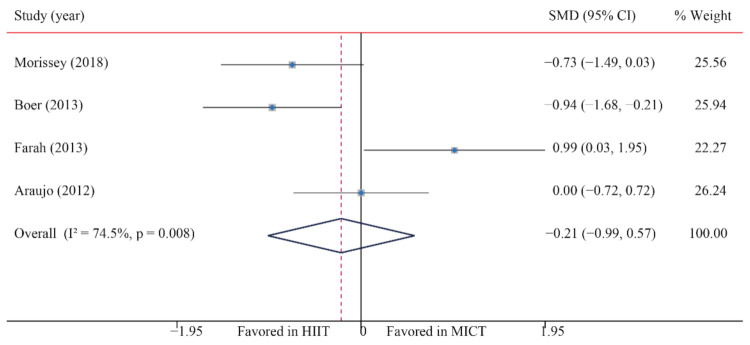
Effect of HIIT versus MICT on blood insulin.

**Figure 16:**
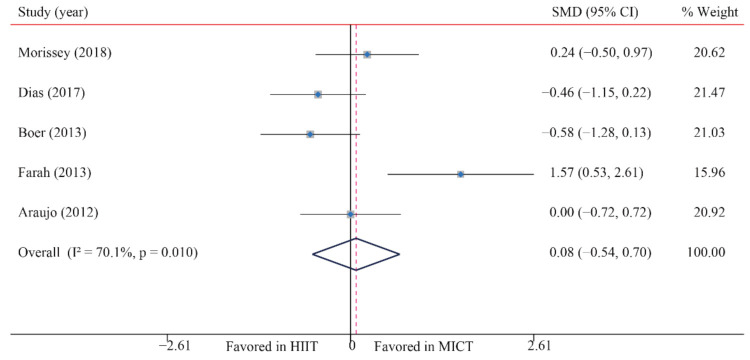
Effect of HIIT versus MICT on HOMA-IR.

**Table 1:** Descriptive characteristics of the included studies of overweight and obese children and adolescents.

Study	Subjects’ Characteristic				Training Information					Outcomes
	Status	Age (Years)	*n*	Gender (M/F)	Group	Training Modality, Setting and Protocol	Time (Min/Session)	Frequency (Times/Week)	Duration (Weeks)	
Miguet et al. [16] France, 2020	OW	13.6 ± 1.5	22	NR	HIIT	Bicycle (lab) 15 × [30 s (90% VO_2max_): 30 s (50 w)]	15	2	16	①
		13.6 ± 1.5	21	NR	MICT	Bicycle (lab) 45 min (70% VO_2max_)	45			
Morissey et al. [17] France, 2018	OW	15.0 ± 1.4	16	4/12	HIIT	Mix (lab) 4–6 × [120–150 s (90–95% HR_max_): 90 s (55% HR_max_)]	14–24	3	12	①③④⑤
		15.0 ± 1.6	13	4/9	MICT	Mix (lab) 40–60 min (65–70% HR_max_)	40–60			
Cvetković et al. [20] Serbia, 2018	OW/OB	11–13	11	11/0	HIIT	Run (school) 3 × 5–10 × [10–20 s (100% MAS): 10–20 s (0)]: 3 min (0)	11–26	3	12	①②③
		11–13	10	10/0	MICT	Football (school) 60 min (NR)	60			
Dias et al. [18] Australia, 2017	OB	7–16	17	NR	HIIT	Treadmill (lab) 4 × [4 min (85–95% HR_max_): 3 min (50–70% HR_max_)]	28	3	12	①②④⑤
		7–16	24	NR	MICT	Treadmill (lab) 44 min (60–70% HR_max_)	44			
Lazzer et al. [27] Italy, 2016	OB	16.8 ± 0.7	10	10/0	HIIT	Treadmill (lab) 6 × [40 s (100% VO_2max_): 5 min (40% VO_2max_)]	34	3	3	①②
		16.4 ± 1.1	11	11/0	MICT	Treadmill (lab) 45 min × (70% VO_2max_)	45			
Kargarfard et al. [19] Iran, 2016	OB	12.4 ± 1.3	10	10/0	HIIT	Treadmill (lab) 8 × [4 min (80–90% HRR): 2 min (40–50% HRR)]	48	3	8	①②③
		12.4 ± 1.3	10	10/0	MICT	Treadmill (lab) 45 min (60–70% HRR)	45			
Murphy et al. [28] USA, 2015	OB	13.7 ± 2.0	7	2/5	HIIT	Treadmill (lab) 10 × [1 min (80–90% HR_max_): 2 min (60% HR_max_)]	30	3	4	①②
		14.3 ± 1.2	6	5/1	MICT	Treadmill (lab) 30 min (65% HR_max_)	30			
Starkoff et al. [29] USA, 2014	OB	14.9 ± 1.6	14	5/9	HIIT	Bicycle (lab) 10 × [2 min (90–95% HR_max_): 1 min (55% HR_max_)]	30	3	6	①②
		14.5 ± 1.4	13	5/8	MICT	Bicycle (lab) 30 min (65–70% HR_max_)	30			
Farah et al. [30] Brazil, 2013	OB	15.4 ± 0.4	9	5/4	HIIT	Treadmill (lab) NR × [30 s (120% MAS): 30 s (0)] EE 350 kcal	NR	3	24	①②③⑤
		14.8 ± 0.4	10	5/5	MICT	Treadmill (lab) NR (80% VT) EE 350 kcal	NR			
Boer et al. [31] Belgium, 2013	OB	18.0 ± 3.2	17	11/6	HIIT	Bicycle (lab) 10 × [15 s (100% VT): 45 s (50% VT)]	10	2	15	①③④⑤
		16.7 ± 3.6	15	10/5	MICT	Bicycle (lab) 30 min (NR)	30			
Koubaa et al. [32] Tunisia, 2013	OB	13.0 ± 0.8	14	14/0	HIIT	Treadmill (lab) NR × [2 min (80–90% MAS): 1 min (0)]	NA	3	12	①②③④
		12.9 ± 0.5	15	15/0	MICT	Treadmill (lab) NR (60–70% MAS)	NA			
Araujo et al. [33] Brazil, 2012	OB	10.7 ± 0.7	15	5/10	HIIT	Bicycle (lab) 3–6 × [1 min (100% MAS): 3 min (50% MAS)]	12–24	3	12	①③④⑤
		10.4 ± 0.9	15	4/11	MICT	Treadmill (lab) 30–60 min (80% HR_max_)	30–60			

Note: ① body composition markers (BM, BMI, BF%, WC, VAT, FFM, etc.), ② cardiorespiratory fitness (VO_2max_, includes yo-yo test distance or PACER times), ③ blood pressure (SBP and DBP), ④ lipid profile (TG, TC, HDL-C, LDL-C, etc.) and ⑤ glucose markers (BG, BI, HOMA-IR). Description of HIIT protocol: 2 × 10 × [1 min (90% HR_max_):1 min (0)]:3 min (0) means 2 sets in each session, 3 min recovery between sets and perform 10 bouts of 1 min work at 90% HR_max_ and 1 min rest each set. BM: body mass, BMI: body mass index, BF%: body fat percentage, BG: blood glucose, BI: blood insulin, DBP: diastolic blood pressure, EE: energy expenditure, FFM: fat-free mass, HDL-C: high-density lipoprotein cholesterol, HIIT: high-intensity interval training, HOMA-IR: homeostasis model assessment, HR_max_: maximal heart rate, LDL-C: low-density lipoprotein cholesterol, MAP: maximal aerobic power, MAS: maximal aerobic speed, MICT: moderate-intensity continuous training, MS: maximal speed, NA: not available, NR: not reported, OB: obese, OW: overweight, SBP: systolic blood pressure, TC: total cholesterol, TG: triglycerides, VAT: visceral adipose tissue, VT: ventilatory threshold, WC: waist circumference, VO_2max_: maximal oxygen uptake.

**Table 2:** Risk of bias assessment of the included studies.

Study	Assessment								Score	Risk of Bias
	1	2	3	4	5	6	7	8		
Miguet	●	●	●	○	○	●	●	○	5	Moderate
Morissey	●	●	●	○	●	●	○	●	6	Moderate
Cvetković	●	●	●	○	●	●	○	●	6	Moderate
Dias	●	●	●	○	●	●	●	●	7	Low
Lazzer	●	●	●	○	●	●	○	●	6	Moderate
Kargarfard	●	●	●	○	●	●	○	●	6	Moderate
Murphy	●	●	●	○	●	●	○	○	5	Moderate
Starkoff	●	●	●	○	●	●	●	○	6	Moderate
Farah	●	●	●	●	○	○	○	●	5	Moderate
Boer	●	○	○	○	●	●	○	○	3	High
Koubaa	●	●	○	○	○	●	○	○	3	High
Araujo	●	●	●	○	●	●	○	●	6	Moderate

Note: (1) Qualification criteria were specified, (2) participants were randomly assigned, (3) there was no significant difference in the baseline values of the main outcome(s) between groups, (4) blinding was used by assessors who measured the main outcome(s), (5) used “intention to treat” to analyze the primary outcome(s) data, (6) reported the dropout of main outcome(s) and the dropout of participants was <20%, (7) calculated the sample size and the study had enough power to detect changes in the main outcome(s) and (8) reported the summary results of each group and estimated effect size (difference between groups) and its precision (e.g., 95% confidence interval). ●: clearly described; ○: absent or unclear.

**Table 3:** Subgroup analysis of effects of HIIT vs. MICT on cardiorespiratory fitness.

Subgroup	Synthesis Studies/Total (%)	SMD (95% CI)	MICT	HIIT	*I*^2^ (%)	*p*-Value	*p* for Interaction
***Total***	8/8 (100)	0.87 (0.39, 1.35)	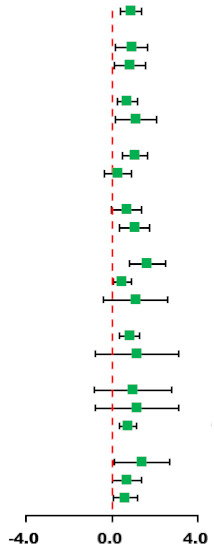		56.8	0.023	
*Gender*					0.903		
Boys	4/8 (50)	0.91 (0.17, 1.65)			63.6	0.041	
Both	4/8 (50)	0.83 (0.10, 1.56)			61.8	0.049	
*Age*					0.472		
≤13 years	4/8 (50)	0.69 (0.23, 1.16)			25.6	0.258	
>13 years	4/8 (50)	1.12 (0.15, 2.09)			73.4	0.010	
*Modality*					0.257		
Running	6/8 (75)	1.05 (0.47, 1.64) *			61.2	0.024	
Cycling	2/8 (25)	0.29 (−0.34, 0.92)			0.0	0.370	
*Duration*					0.525		
≤8 weeks	4/8 (50)	0.68 (−0.04, 1.39)			57.3	0.071	
>8 weeks	4/8 (50)	1.05 (0.34, 1.76) *			63.1	0.043	
*Time*					0.473		
<20 min	2/8 (25)	1.64 (0.79, 2.49) *			34.2	0.218	
20–30 min	4/8 (50)	0.47 (0.06, 0.88) *			0.0	0.633	
>30 min	2/8 (25)	1.09 (−0.40, 2.58)			79.0	0.029	
*Settings*					0.375		
Laboratory	6/8 (75)	0.81 (0.34, 1.28) *			44.0	0.112	
School	2/8 (25)	1.16 (−0.78, 3.10)			86.1	0.007	
*Work/rest ratio*					0.686		
<1	2/8 (25)	0.98 (−0.81, 2.76)			82.1	0.018	
=1	2/8 (25)	1.16 (−0.78, 3.10)			86.1	0.007	
>1	4/8 (50)	0.75 (0.36, 1.14) *			0.1	0.391	
*Work duration*					0.296		
<1 min	3/8 (38)	1.38 (0.10, 2.66) *			79.0	0.009	
1–4 min	2/8 (24)	0.68 (−0.01, 1.37)			46.4	0.155	
=4 min	3/8 (38)	0.62 (0.08, 1.17) *			0.0	0.446	
			

* means that the improvement of VO_2max_ is significantly different in this subgroup parameter.

**Table 4:** Subgroup analysis of effects of HIIT vs. MICT on systolic blood pressure.

Subgroup	Synthesis Studies/Total (%)	SMD (95% CI)	MICT	HIIT	*I*^2^ (%)	*p*-Value	*p* for Interaction
***Total***	7/7 (100)	−0.64 (−1.05, −0.22)	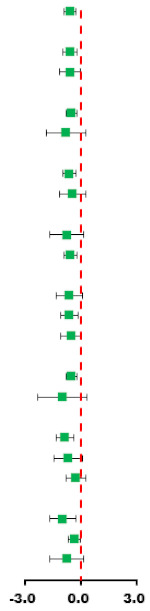		44.9	0.092	
*Gender*					0.770		
Boys	4/7 (51)	−0.58 (−0.98, −0.18) *			0.0	0.009	
Both	3/7 (49)	−0.78 (−1.83, 0.27)			79.0	0.072	
*Age*					0.770		
≤13 years	4/7 (51)	−0.58 (−0.98, −0.18) *			0.0	0.722	
>13 years	3/7 (49)	−0.78 (−1.83, 0.27)			79.0	0.009	
*Modality*					0.489		
Running	5/7 (71)	−0.73 (−1.16, −0.31) *			20.2	0.286	
Cycling	2/7 (29)	−0.40 (−1.54, 0.75)			79.5	0.027	
*Duration*					0.876		
≤8 weeks	1/7 (14)	−0.74 (−1.65, 0.17)			-	-	
>8 weeks	6/7 (86)	−0.63 (−1.11, −0.15) *			53.7	0.056	
*Time*					0.794		
<20 min	4/7 (71)	−0.66 (−1.38, 0.07)			69.7	0.019	
20–30 min	1/7 (14)	−0.34 (−1.20, 0.52)			-	-	
>30 min	2/7 (25)	−0.82 (−1.40, −0.24) *			44.9	0.092	
*Settings*					0.493		
Laboratory	5/7 (71)	−0.54 (−0.98, −0.11) *			36.9	0.175	
School	2/7 (29)	−0.98 (−2.31, 0.35)			73.4	0.052	
*Work/rest ratio*					0.169		
<1	2/7 (29)	−0.93 (−1.46, −0.40) *			0.0	0.843	
=1	2/7 (29)	−0.98 (−2.31, 0.35)			73.4	0.052	
>1	3/7 (42)	−0.26 (−0.78, 0.26)			23.4	0.271	
*Work duration*					0.493		
<1 min	3/7 (43)	−0.96 (−1.65, −0.26) *			47.5	0.149	
1–4 min	3/7 (43)	−0.35 (−0.95, 0.25)			49.4	0.138	
=4 min	1/7 (14)	−0.74 (−1.65, 0.17)			-	-	
			

* means that the improvement of VO_2max_ is significantly different in this subgroup parameter.

**Table 5:** Effects of HIIT and MICT on body composition, cardiorespiratory fitness, blood pressure and glycolipid metabolism indicators.

Outcomes	Studies/Total (%)	*N*	SMD (95% CI)	Favored HIIT	Favored MICT	*I* ^2^	*Q*	*p*-Value
***Total***	12/12 (100)	325		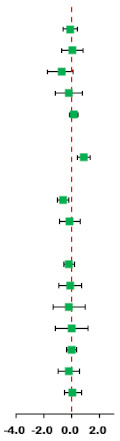				
*Body composition*								
BM	11/12 (92)	313	−0.10 (−0.61, 0.41)			78.4	46.3	0.001
BMI	12/12 (100)	325	0.03 (−0.75, 0.80)			89.8	107.3	0.001
BF%	10/12 (83)	277	−0.23 (−0.73, 0.28)			74.5	35.3	0.001
AF	6/12 (50)	178	−0.21 (−1.19, 0.76)			88.3	42.8	0.001
FFM	6/12 (50)	184	0.15 (−0.16, 0.45)			3.6	5.2	0.394
*Cardiorespiratory fitness*								
VO_2max_	8/12 (67)	191	0.87 (0.39, 1.35) *			56.8	16.2	0.023
*Blood pressure*								
SBP	7/12 (58)	180	−0.64 (−1.05, −0.22) *			44.9	10.9	0.092
DBP	7/12 (58)	180	−0.13 (−0.88, 0.61)			82.1	33.6	0.001
*Glycolipid metabolism*								
TG	5/12 (42)	161	−0.20 (−0.59, 0.19)			33.2	6.0	0.200
TC	5/12 (42)	161	−0.11 (−0.94, 0.72)			83.9	24.8	0.001
HDL-C	4/12 (33)	125	−0.18 (−1.34, 0.99)			89.5	28.5	0.001
LDL-C	3/12 (25)	101	−0.01 (−1.19, 1.18)			87.0	15.4	0.001
BG	4/12 (33)	125	−0.02 (−0.37, 0.34)			0	2.9	0.406
BI	4/12 (33)	125	−0.21 (−0.99, 0.57)			74.5	11.8	0.008
HOMA-IR	4/12 (33)	132	0.08 (−0.54, 0.70)			70.1	13.4	0.010
						

Note: VO_2max_, FFM and HDL-C were positively correlated with health benefits; therefore, the forest plot reflects that the favorable direction of these two indicators was opposite to the labeling direction, that is, HIIT is shown as favorable on the right side of the invalid line. * means that the improvement of VO_2max_ is significantly different in this subgroup parameter.

## Supplementary Materials

The following are available online at www.mdpi.com/1660-4601/182/21/1905/s1, Figure S1. Sensitivity analysis of high-intensity interval training versus moderate-intensity continuous training on body mass (BM)—HIIT vs. MICT metainf; Figure S2. Sensitivity analysis of high-intensity interval training versus moderate-intensity continuous training on body mass index (BMI)—HIIT vs. MICT metainf.; Figure S3. Sensitivity analysis of high-intensity interval training versus moderate-intensity continuous training on body fat percentage (BF%)—HIIT vs. MICT metainf; Figure S4. Sensitivity analysis of high-intensity interval training versus moderate-intensity continuous training on abdominal fat (AF)—HIIT vs. MICT metainf, Figure S5. Sensitivity analysis of high-intensity interval training versus moderate-intensity continuous training on fat free mass (FFM)—HIIT vs. MICT metainf; Figure S6. Sensitivity analysis of high-intensity interval training versus moderate-intensity continuous training on maximal oxygen uptake (VO_2max_)—HIIT vs. MICT metainf; Figure S7. Sensitivity analysis of high-intensity interval training versus moderate-intensity continuous training on systolic blood pressure (SBP)—HIIT vs. MICT metainf; Figure S8. Sensitivity analysis of high-intensity interval training versus moderate-intensity continuous training on diastolic blood pressure (DBP)—HIIT vs. MICT metainf; Figure S9. Sensitivity analysis of high-intensity interval training versus moderate-intensity continuous training on blood glucose (BG)—HIIT vs. MICT metainf; Figure S10. Sensitivity analysis of high-intensity interval training versus moderate-intensity continuous training on blood insulin (BI)—HIIT vs. MICT metainf; Figure S11. Sensitivity analysis of high-intensity interval training versus moderate-intensity continuous training on HOMA-IR—HIIT vs. MICT metainf; Figure S12. Sensitivity analysis of high-intensity interval training versus moderate-intensity continuous training on triglycerides (TG)—HIIT vs. MICT metainf; Figure S13. Sensitivity analysis of high-intensity interval training versus moderate-intensity continuous training on total cholesterol (TC)—HIIT vs. MICT metainf; Figure S14. Sensitivity analysis of high-intensity interval training versus moderate-intensity continuous training on high-density lipoprotein cholesterol (HDL-C)—HIIT vs. MICT metainf; Figure S15. Sensitivity analysis of high-intensity interval training versus moderate-intensity continuous training on low-density lipoprotein cholesterol (LDL-C)—HIIT vs. MICT metainf; Figure S16. Funnel plot of high-intensity interval training versus moderate-intensity continuous training on body mass (BM)—HIIT vs. MICT plot and metatrim results; Figure S17. Funnel plot of high-intensity interval training versus moderate-intensity continuous training on body mass index–HIIT vs. MICT plot and metatrim results; Figure S18. Funnel plot of high-intensity interval training versus moderate-intensity continuous. Funnel plot of high-intensity interval training versus moderate-intensity continuous training on fat free mass; Figure S19. Funnel plot of high-intensity interval training versus moderate-intensity continuous. Funnel plot of high-intensity interval training versus moderate-intensity continuous training on maximal oxygen uptake (VO_2max_)—HIIT vs. MICT plot and metatrim results; Figure S20. Funnel plot of high-intensity interval training versus moderate-intensity continuous. Results of trim and fill method for high-intensity interval training versus moderate-intensity continuous training on systolic blood pressure (SBP)—HIIT vs. MICT plot and metatrim results; Figure S21. Funnel plot of high-intensity interval training versus moderate-intensity continuous. Results of trim and fill method for high-intensity interval training versus moderate-intensity continuous training on diastolic blood pressure (DBP); Figure S22. Funnel plot of high-intensity interval training versus moderate-intensity continuous training on triglycerides (TG)—HIIT vs. MICT plot and metatrim results; Figure S23. Funnel plot of high-intensity interval training versus moderate-intensity continuous. Funnel plot of high-intensity interval training versus moderate-intensity continuous training on high-density lipoprotein cholesterol (HDL); Figure S24. Funnel plot of high-intensity interval training versus moderate-intensity continuous training on low-density lipoprotein cholesterol (LDL)—HIIT vs. MICT plot and metatrim results.; Figure S25. Funnel plot of high-intensity interval training versus moderate-intensity continuous training on blood glucose (BG)—HIIT vs. MICT plot and metatrim results; Figure S26. Funnel plot of high-intensity interval training versus moderate-intensity continuous. Funnel plot of high-intensity interval training versus moderate-intensity continuous training on homeostasis model assessment (HOMA-IR)—HIIT vs. MICT plot and metatrim results.
