# Supplementary material for: Effects of High-Intensity Interval Training and Moderate-Intensity Continuous Training on Cardiometabolic Risk Factors in Overweight and Obesity Children and Adolescents: A Meta-Analysis of Randomized Controlled Trials
Source: Int J Environ Res Public Health. 2021 Nov 12;18(22):11905. doi: 10.3390/ijerph182211905 (PMC8623248; doi:10.3390/ijerph182211905)
Supplement: Supplementary file 1 [file ijerph-18-11905-s001.zip › ijerph-1408297-supplementary.pdf]

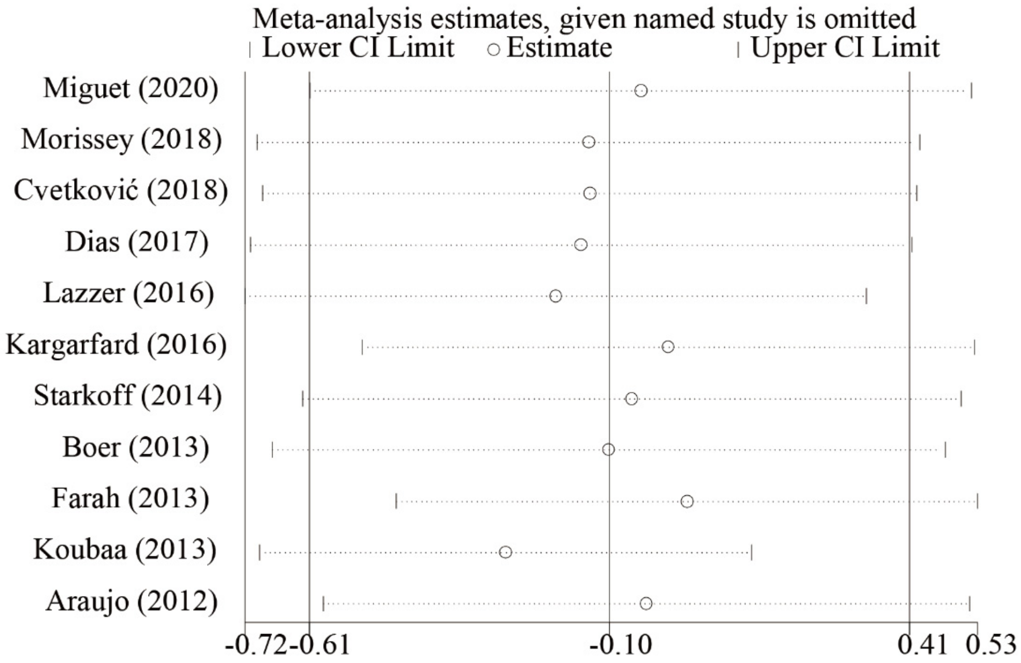

Figure S1. Sensitivity analysis of high-intensity interval training versus moderate-intensity continuous training on body mass (BM)—HIIT vs. MICT metainf

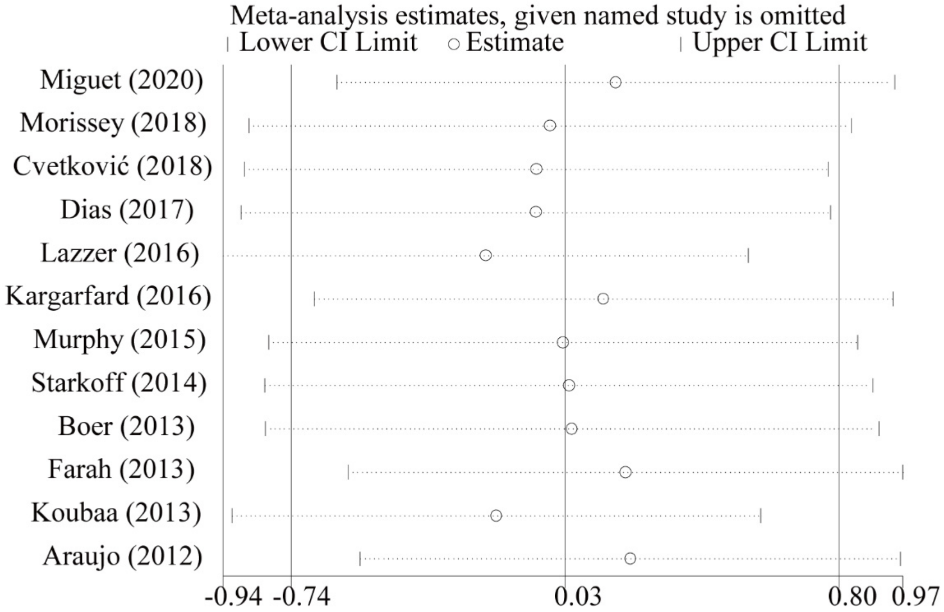

Figure S2. Sensitivity analysis of high-intensity interval training versus moderate-intensity continuous training on body mass index (BMI)—HIIT vs. MICT metainf.

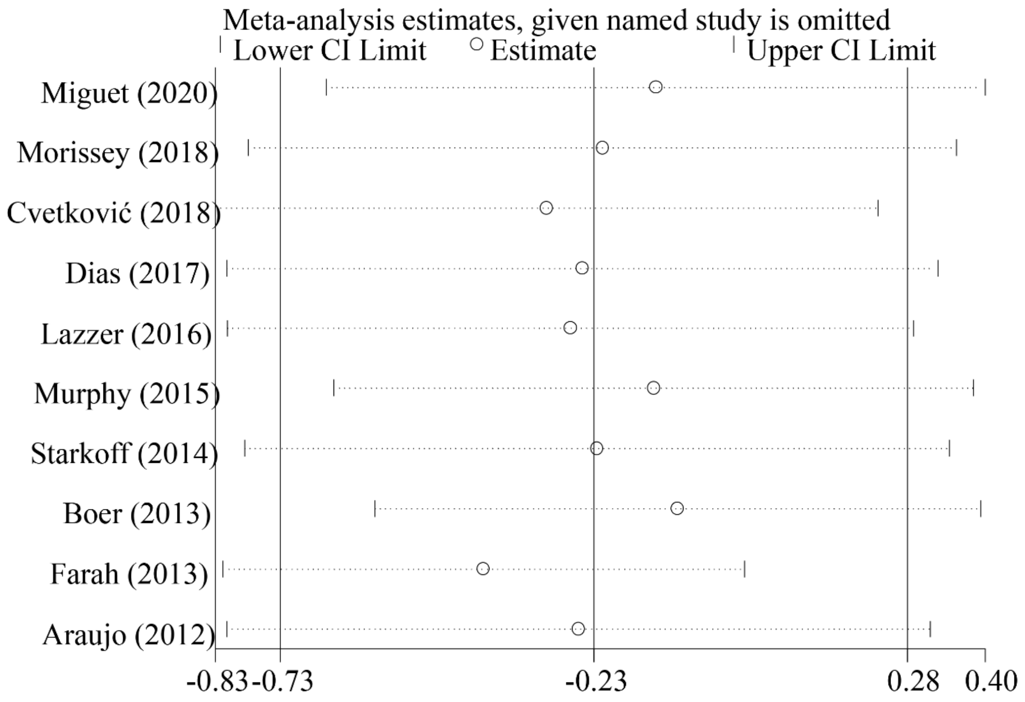

Figure S3. Sensitivity analysis of high-intensity interval training versus moderate-intensity continuous training on body fat percentage (BF%)—HIIT vs. MICT metainf.

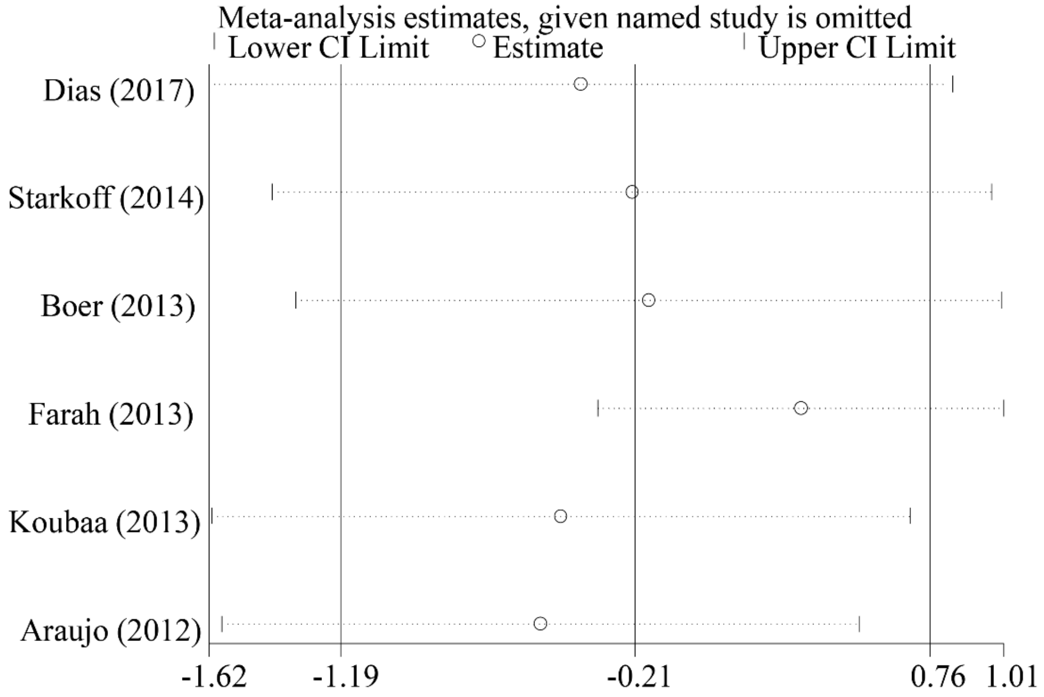

Figure S4. Sensitivity analysis of high-intensity interval training versus moderate-intensity continuous training on abdominal fat (AF)—HIIT vs. MICT metainf.

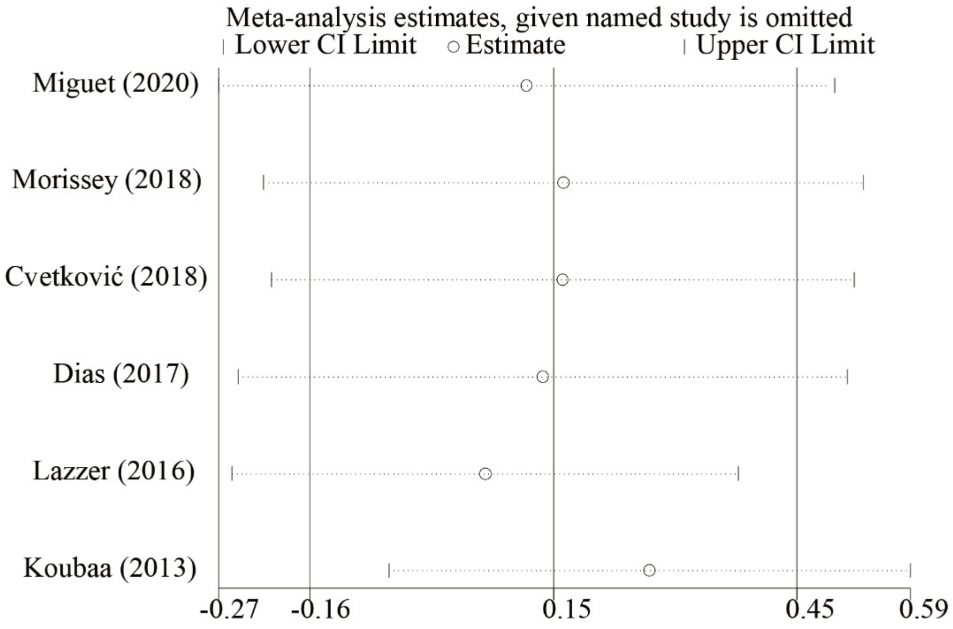

Figure S5. Sensitivity analysis of high-intensity interval training versus moderate-intensity continuous training on fat free mass (FFM)—HIIT vs. MICT metainf.

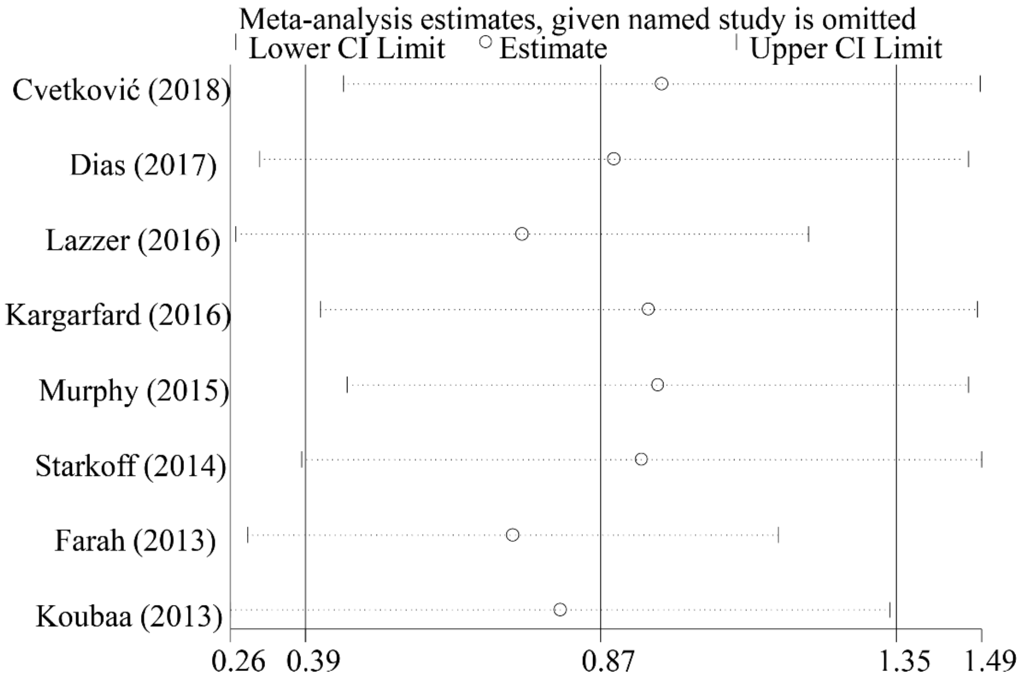

Figure S6. Sensitivity analysis of high-intensity interval training versus moderate-intensity continuous training on maximal oxygen uptake (VO_{2max})—HIIT vs. MICT metainf.

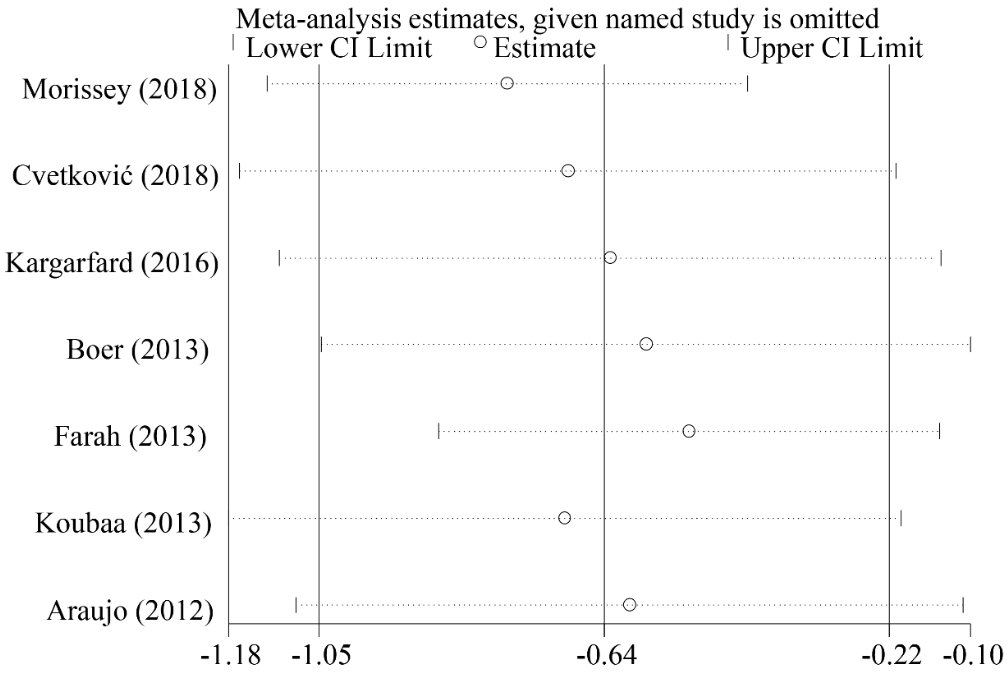

Figure S7. Sensitivity analysis of high-intensity interval training versus moderate-intensity continuous training on systolic blood pressure (SBP)—HIIT vs. MICT metainf.

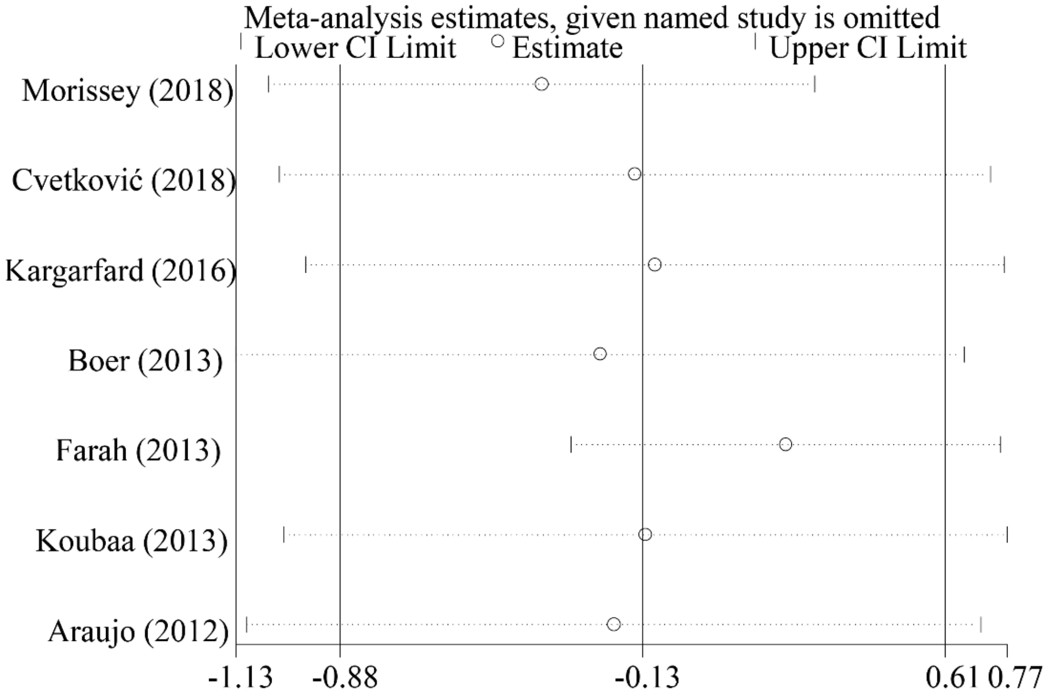

Figure S8. Sensitivity analysis of high-intensity interval training versus moderate-intensity continuous training on diastolic blood pressure (DBP)—HIIT vs. MICT metainf.

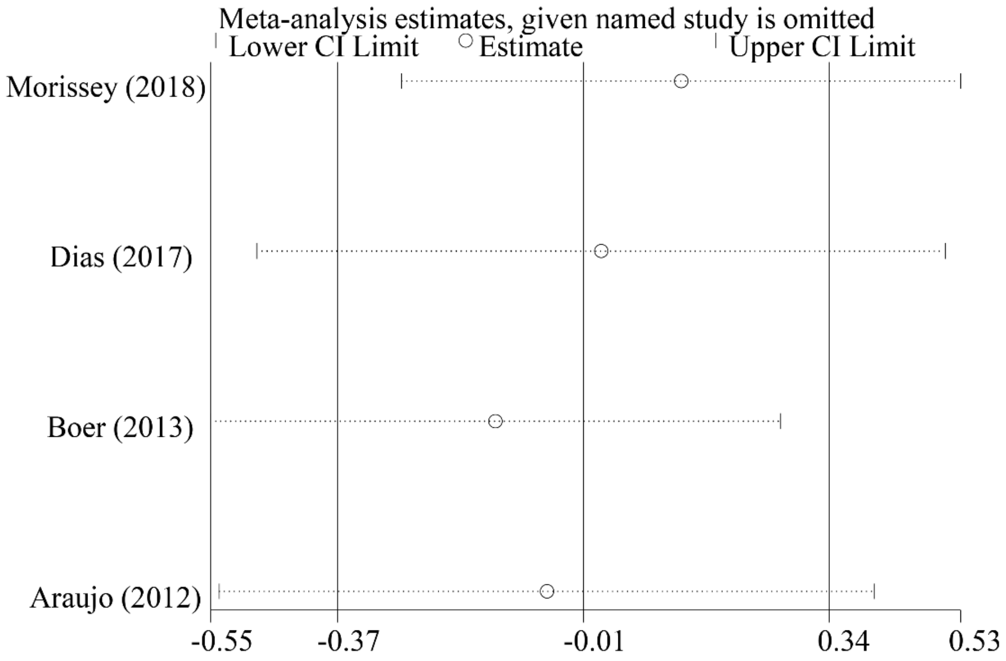

Figure S9. Sensitivity analysis of high-intensity interval training versus moderate-intensity continuous training on blood glucose (BG)—HIIT vs. MICT metainf.

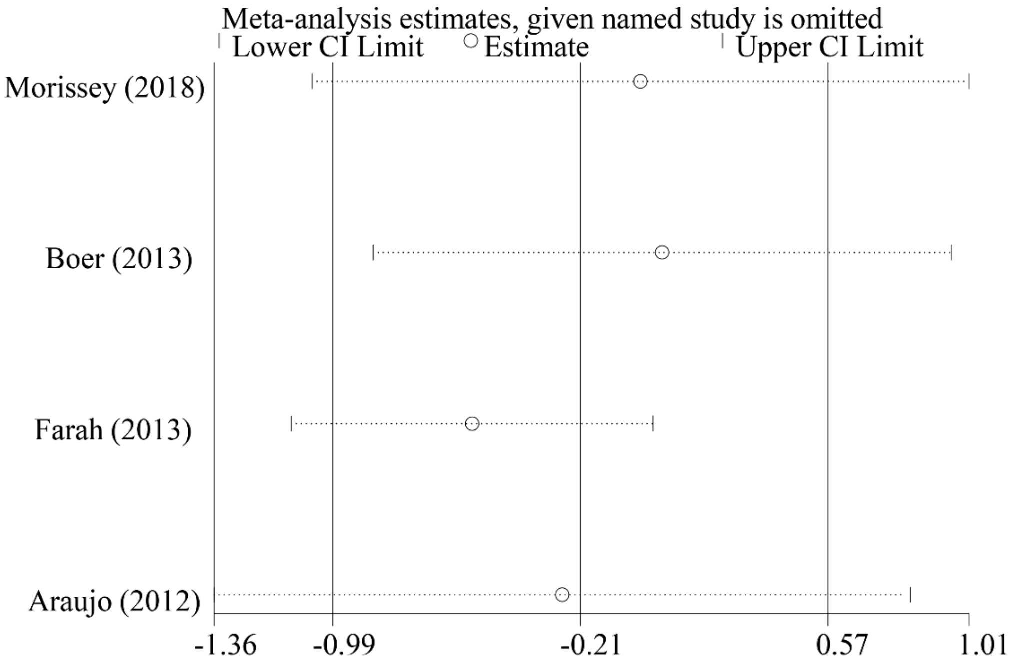

Figure S10. Sensitivity analysis of high-intensity interval training versus moderate-intensity continuous training on blood insulin (BI)—HIIT vs. MICT metainf.

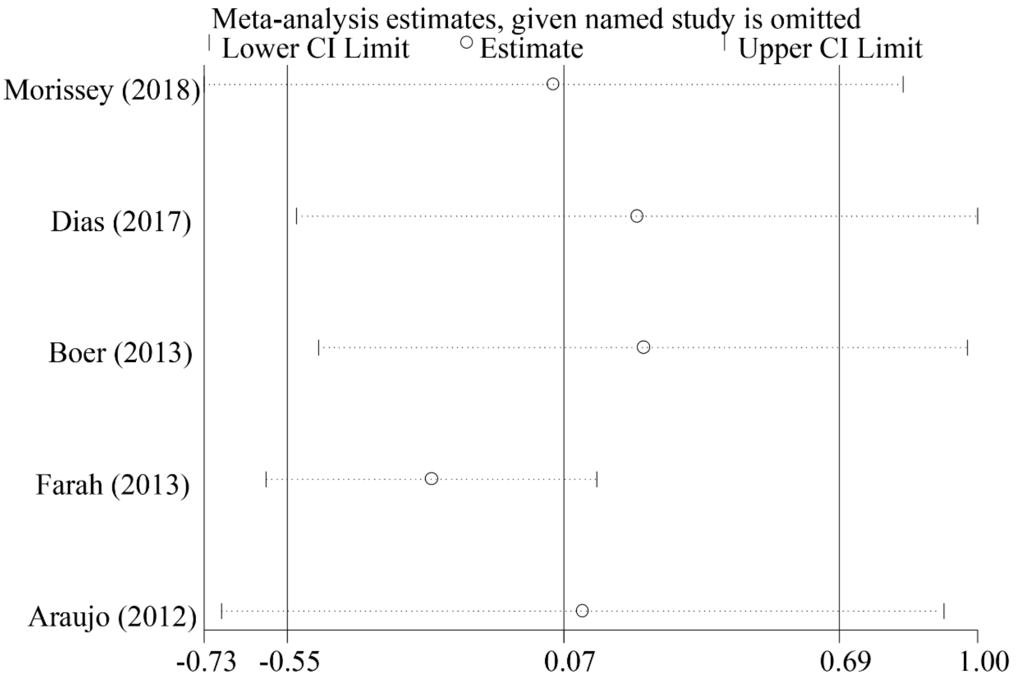

Figure S11. Sensitivity analysis of high-intensity interval training versus moderate-intensity continuous training on HOMA-IR—HIIT vs. MICT metainf.

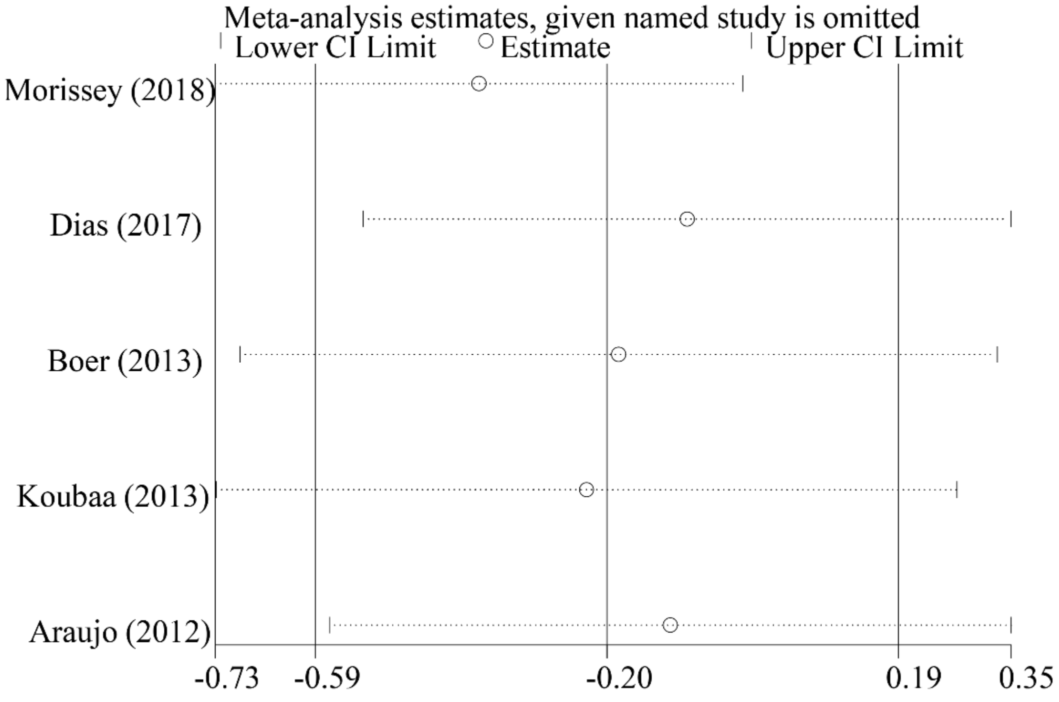

Figure S12. Sensitivity analysis of high-intensity interval training versus moderate-intensity continuous training on triglycerides (TG)—HIIT vs. MICT metainf.

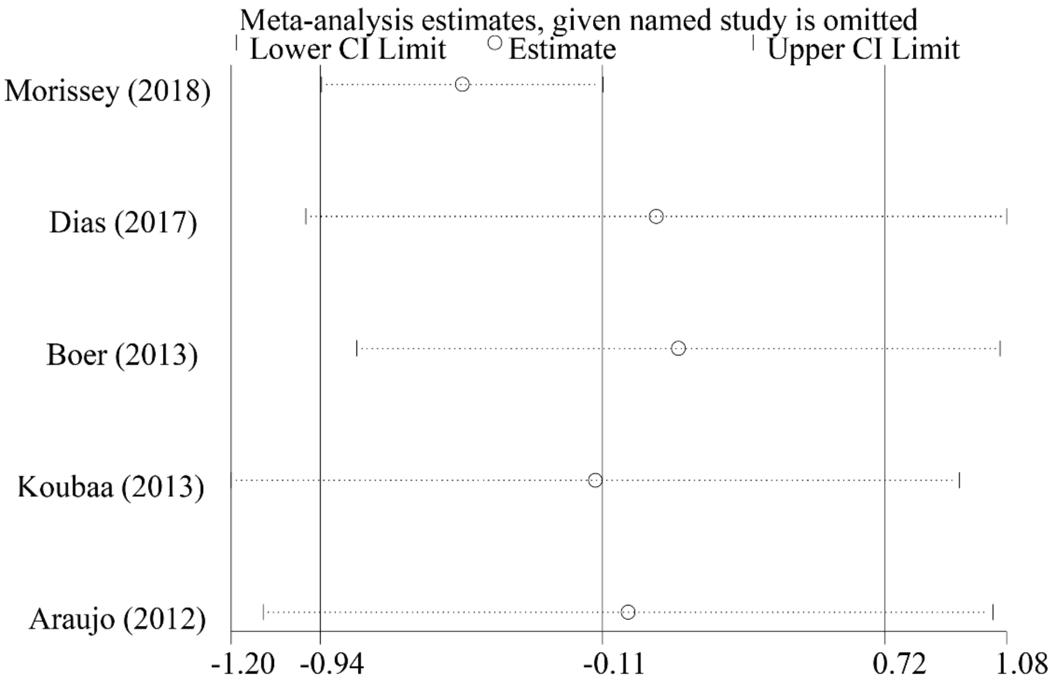

Figure S13. Sensitivity analysis of high-intensity interval training versus moderate-intensity continuous training on total cholesterol (TC)—HIIT vs. MICT metainf.

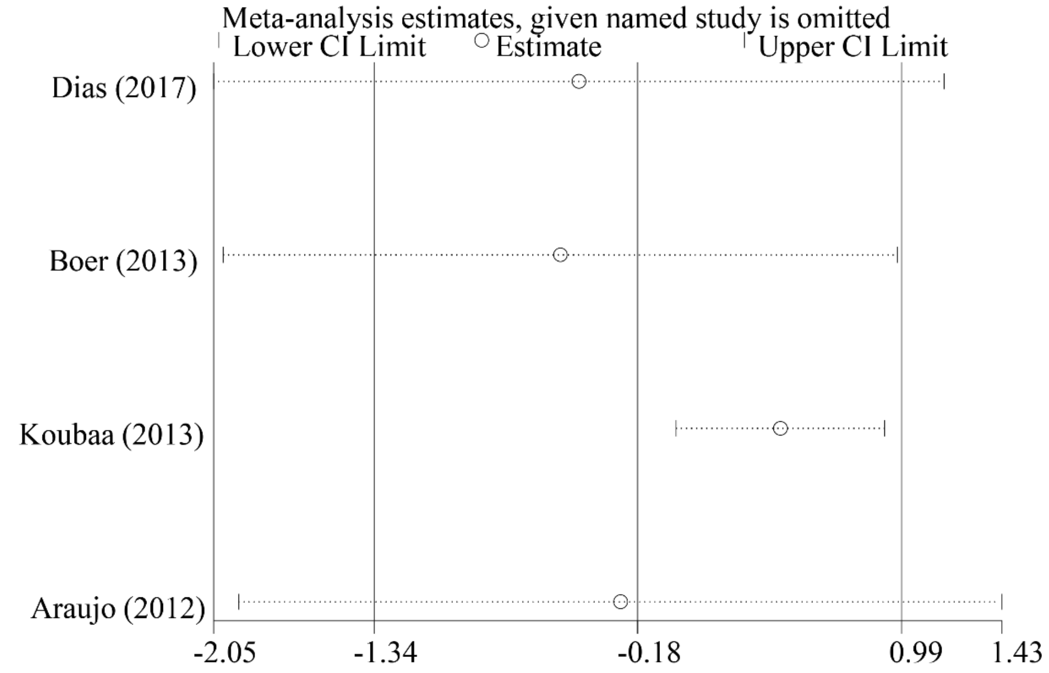

Figure S14. Sensitivity analysis of high-intensity interval training versus moderate-intensity continuous training on high-density lipoprotein cholesterol (HDL-C)—HIIT vs. MICT metainf.

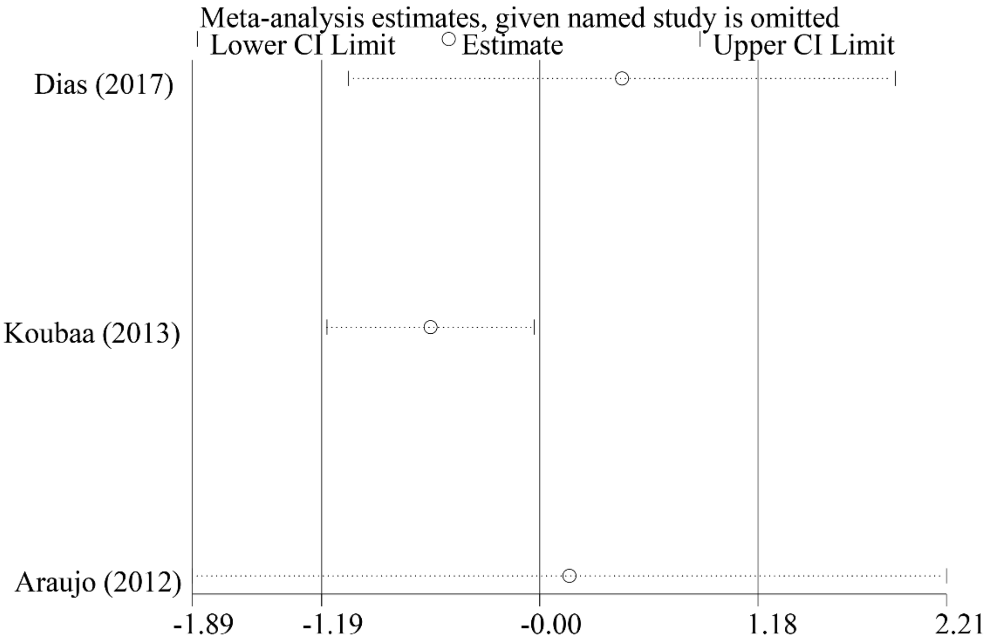

Figure S15. Sensitivity analysis of high-intensity interval training versus moderate-intensity continuous training on low-density lipoprotein cholesterol (LDL-C)—HIIT vs. MICT metainf.

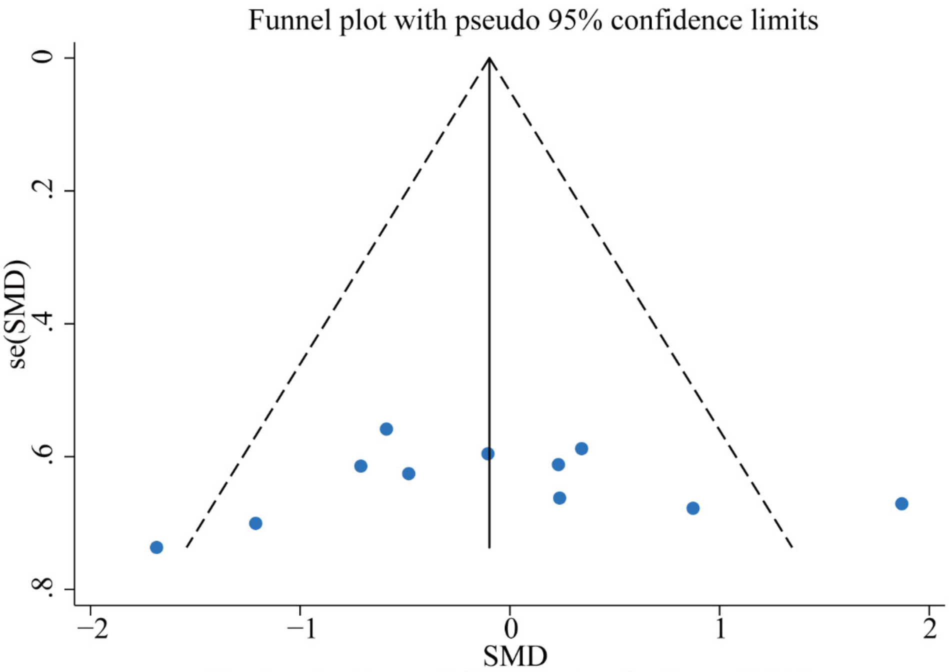

Figure S16. Funnel plot of high-intensity interval training versus moderate-intensity continuous training on body mass（BM)—HIIT vs. MICT plot and metatrim results.

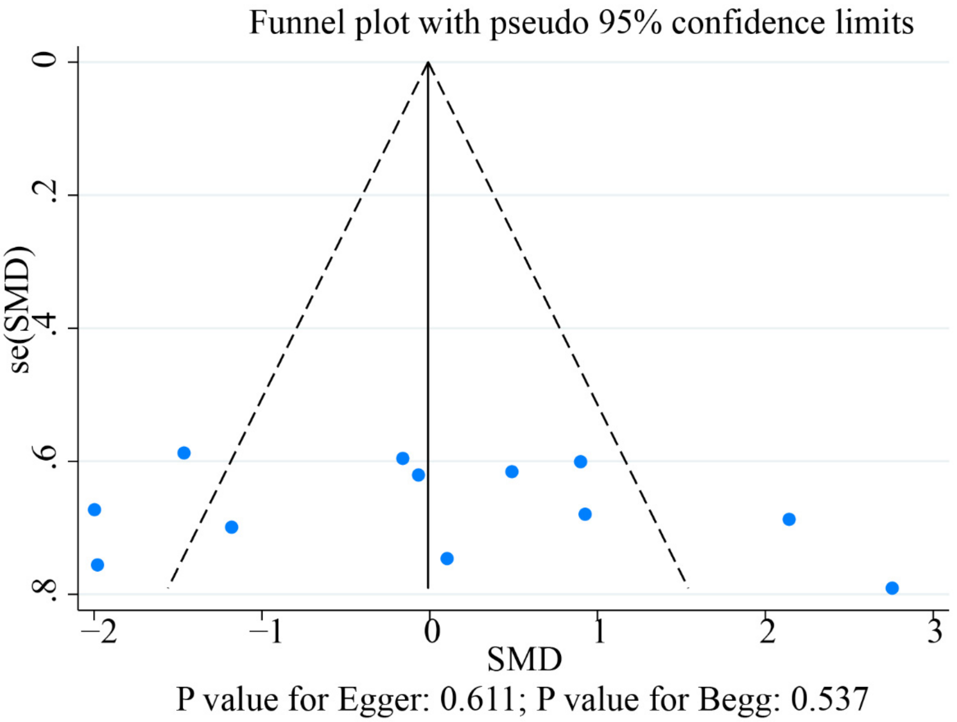

Figure S17. Funnel plot of high-intensity interval training versus moderate-intensity continuous training on body mass index–HIIT vs. MICT plot and metatrim results.

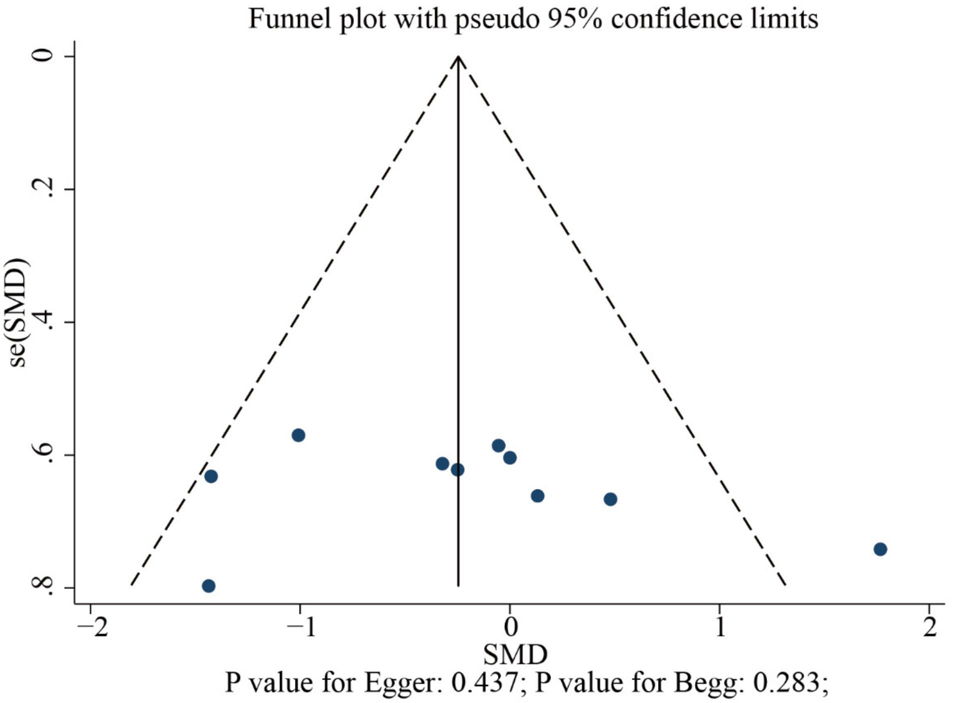

Figure S18. Funnel plot of high-intensity interval training versus moderate-intensity continuous. Funnel plot of high-intensity interval training versus moderate-intensity continuous training on fat free mass.

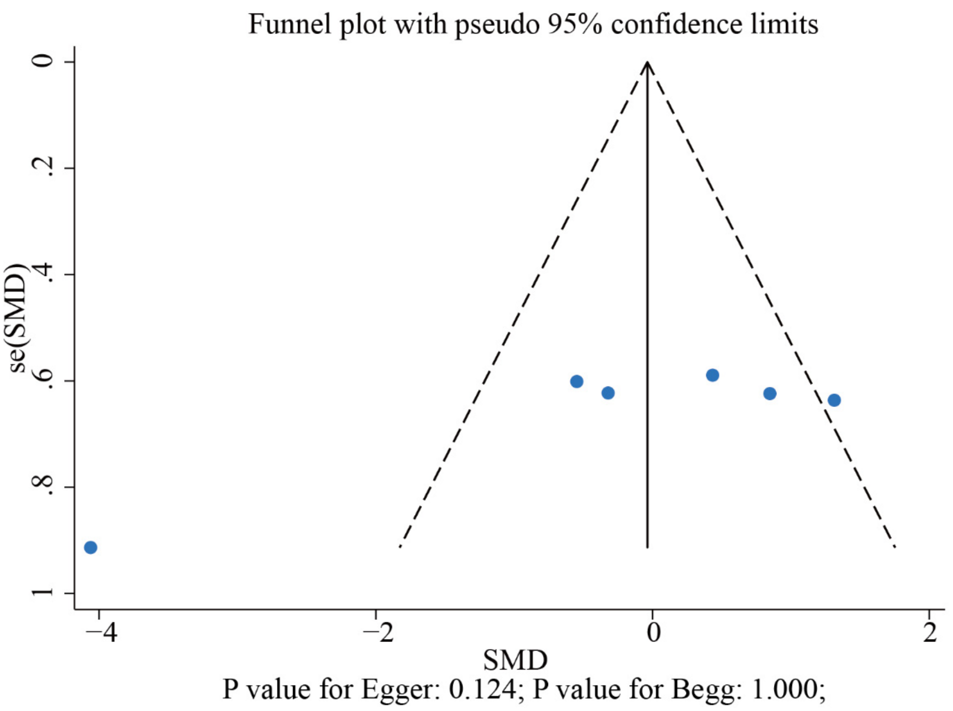

Figure S19. Funnel plot of high-intensity interval training versus moderate-intensity continuous. Funnel plot of high-intensity interval training versus moderate-intensity continuous training on maximal oxygen uptake (VO_{2max})—HIIT vs. MICT plot and metatrim results.

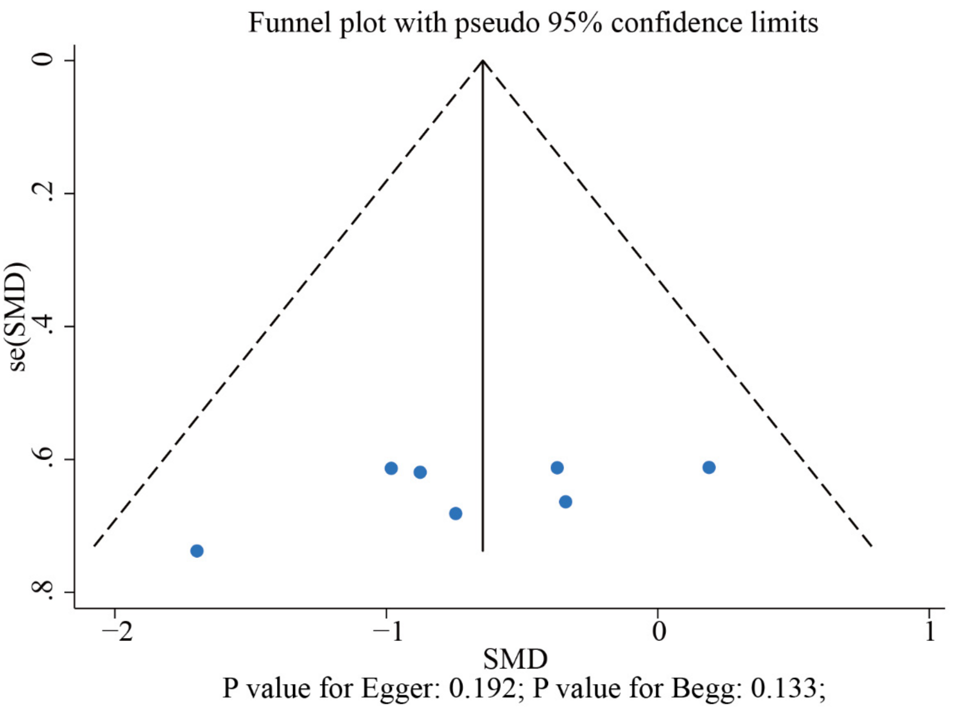

Figure S20. Funnel plot of high-intensity interval training versus moderate-intensity continuous. Results of trim and fill method for high-intensity interval training versus moderate-intensity continuous training on systolic blood pressure (SBP)—HIIT vs. MICT plot and metatrim results.

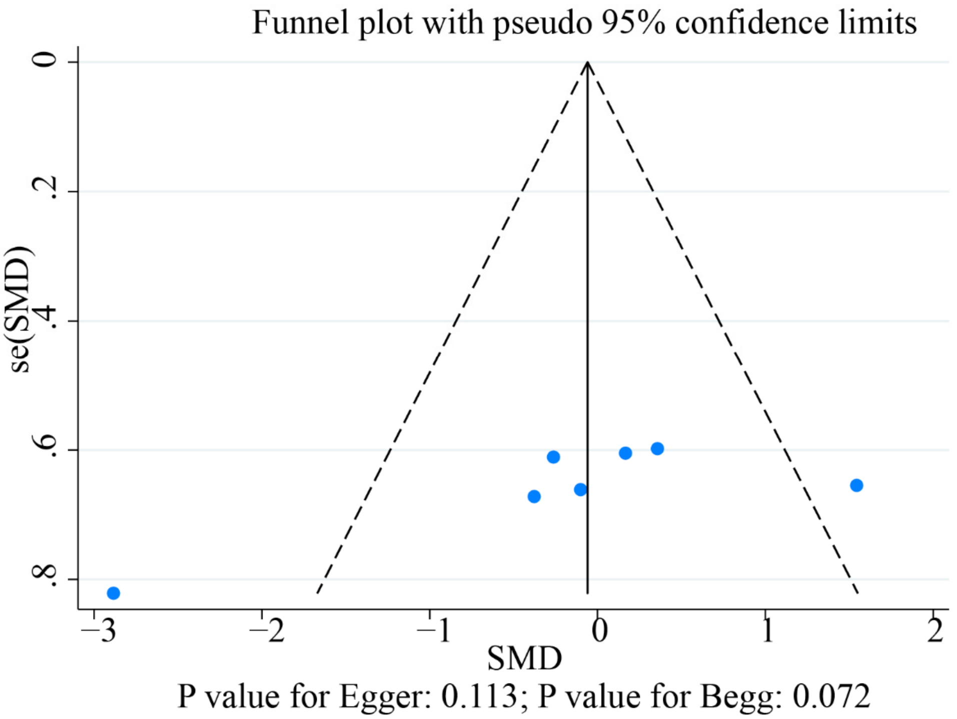

Figure S21. Funnel plot of high-intensity interval training versus moderate-intensity continuous. Results of trim and fill method for high-intensity interval training versus moderate-intensity continuous training on diastolic blood pressure (DBP).

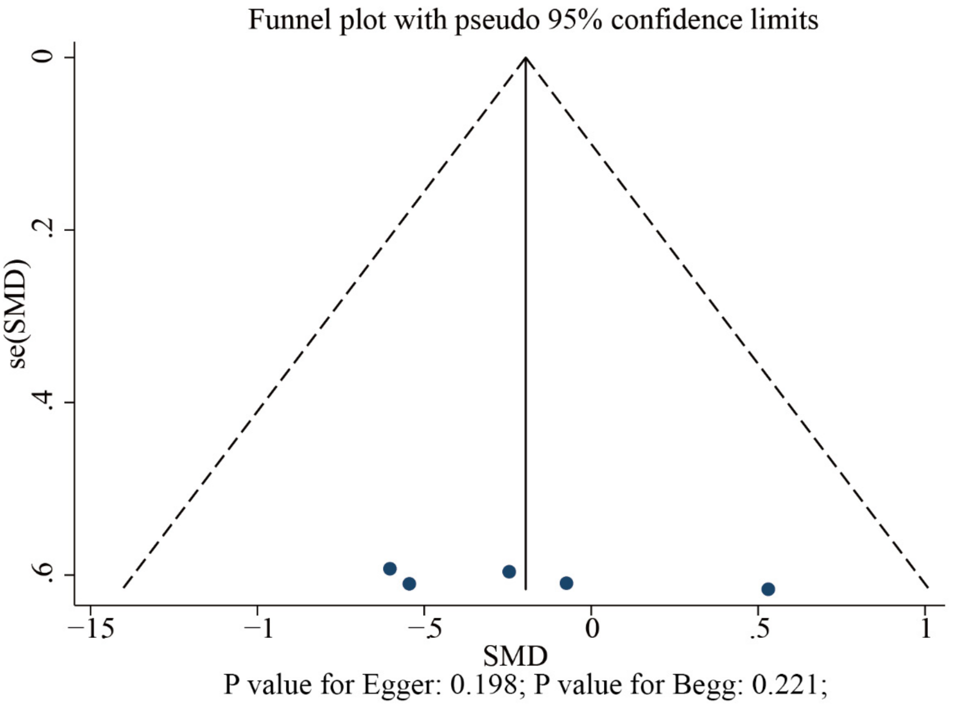

Figure S22. Funnel plot of high-intensity interval training versus moderate-intensity continuous training on triglycerides （TG—HIIT vs. MICT plot and metatrim results.

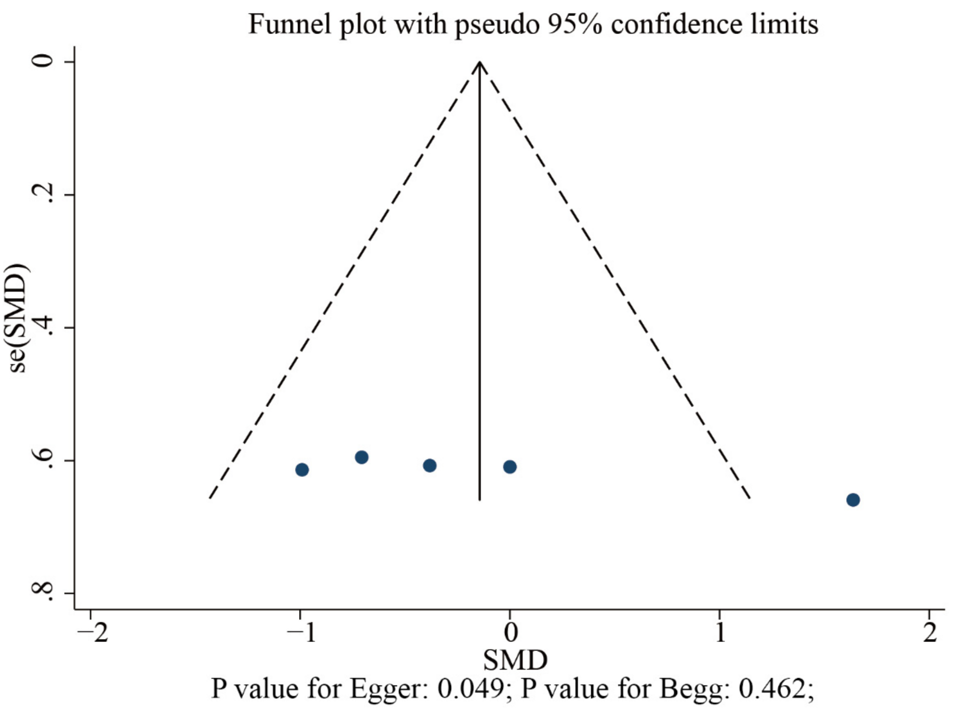

Figure S23. Funnel plot of high-intensity interval training versus moderate-intensity continuous. Funnel plot of high-intensity interval training versus moderate-intensity continuous training on high-density lipoprotein cholesterol（HDL）.

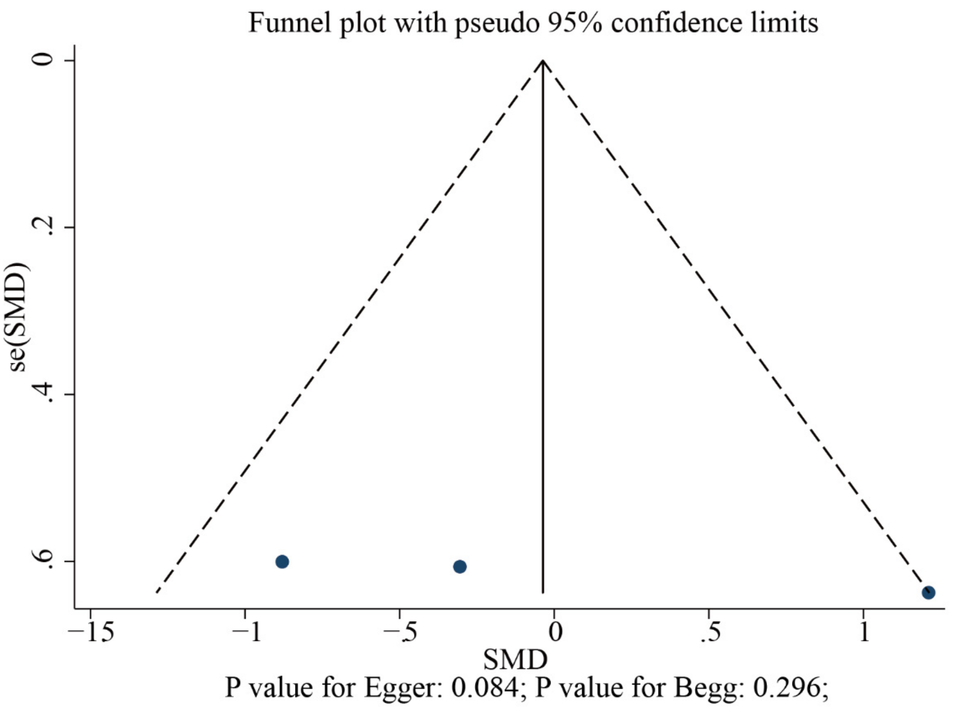

Figure S24. Funnel plot of high-intensity interval training versus moderate-intensity continuous training on low-density lipoprotein cholesterol（LDL）—HIIT vs. MICT plot and metatrim results.

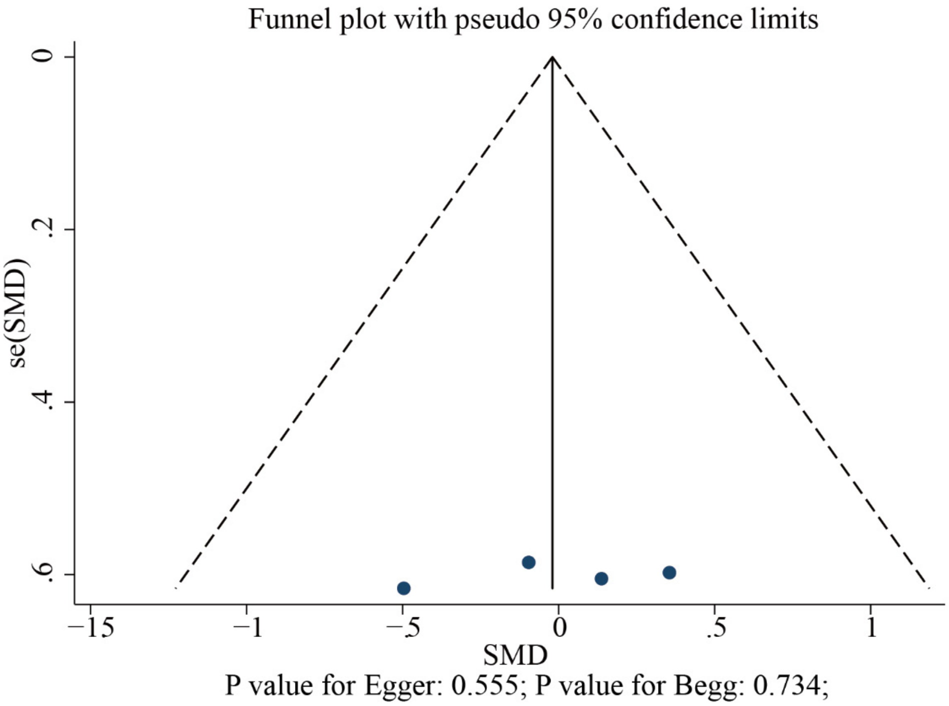

Figure S25. Funnel plot of high-intensity interval training versus moderate-intensity continuous training on blood glucose（BG）—HIIT vs. MICT plot and metatrim results.

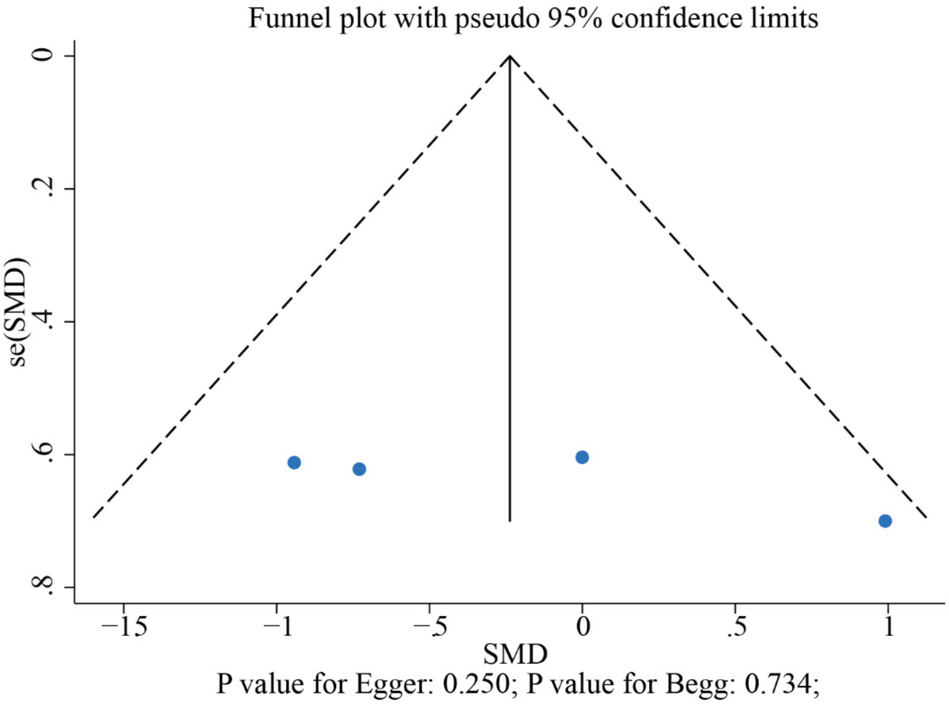

Figure S26. Funnel plot of high-intensity interval training versus moderate-intensity continuous. Funnel plot of high-intensity interval training versus moderate-intensity continuous training on homeostasis model assessment（HOMA-IR）—HIIT vs. MICT plot and metatrim results.
